# Deep generative classification of blood cell morphology

**DOI:** 10.1038/s42256-025-01122-7

**Published:** 2025-11-19

**Authors:** Simon Deltadahl, Julian Gilbey, Christine Van Laer, Nancy Boeckx, Mathie P. G. Leers, Tanya Freeman, Laura Aiken, Timothy Farren, Matthew Smith, Mohamad Zeina, Stephen MacDonald, Daniel Gleghorn, BloodCounts! consortium, Nancy Boeckx, Mathie P. G. Leers, Martijn Schut, Folkert Asselbergs, Sujoy Kar, Sophie Williams, Mickey Koh, Yvonne Henskens, Norbert de Wit, Umberto D’Alessandro, Bubacarr Bah, Ousman Secka, Rajeev Gupta, Sara Trompeter, Christine van Laer, Gordon A. Awandare, Kwabena Sarpong, Lucas Amenga-Etego, Willem H. Ouwehand, James H. F. Rudd, Nicholas Gleadall, Carola-Bibiane Schönlieb, Suthesh Sivapalaratnam, Michael Roberts, Parashkev Nachev, James HF Rudd, Concetta Piazzese, Joseph Taylor, Nicholas Gleadall, Carola-Bibiane Schönlieb, Suthesh Sivapalaratnam, Michael Roberts, Parashkev Nachev

**Affiliations:** 1 Department of Applied Mathematics and Theoretical Physics, University of Cambridge, Cambridge, UK; 2 Department of Laboratory Medicine, UZ Leuven, Leuven, Belgium; 3 Department of Oncology, KU Leuven, Leuven, Belgium; 4 Department of Clinical Chemistry and Hematology, Zuyderland Medical Center, Sittard-Geleen, The Netherlands; 5 Department of Environmental Sciences, Faculty of Science, Open Universiteit, Heerlen, The Netherlands; 6 Department of Clinical Haematology, Royal London Hospital, Barts Health NHS Trust, London, UK; 7 High Dimensional Neurology Group, UCL Queen Square Institute of Neurology, University College London, London, UK; 8 Cambridge University Hospitals NHS Trust, Cambridge, UK; 9 Department of Medicine, University of Cambridge, Cambridge, UK; 10 Barts Life Sciences, Barts Health NHS Trust, London, UK; 11 Precision Healthcare University Research Institute, Queen Mary University of London, London, UK; 12 National Health Service Blood and Transplant, Cambridge, UK; 13 Department of Haematology, Victor Phillip Dahdaleh Heart and Lung Research Institute, University of Cambridge, Cambridge, UK; 14 Department of Clinical Chemistry, Amsterdam UMC, Amsterdam, The Netherlands; 15 Vrije Universiteit Amsterdam, Amsterdam, The Netherlands; 16 Institute of Cardiovascular Science, University College London, London, UK; 17 Apollo Hospitals, Chennai, India; 18 Department of Haematology, St George’s University Hospitals NHS Foundation Trust, London, UK; 19 Health Sciences Authority, Singapore, Singapore; 20 Department of Clinical Chemistry and Haematology, Maastricht University Medical Centre+, Maastricht, The Netherlands; 21 MRC Unit The Gambia at the London School of Hygiene & Tropical Medicine, Banjul, The Gambia; 22 Department of Haematology, University College London Hospitals, London, UK; 23 West African Centre for Cell Biology of Infectious Pathogens, University of Ghana, Accra, Ghana; 24 Department of Haematology, Cambridge University Hospitals, Cambridge Biomedical Campus, Cambridge, UK; 25 Department of Haematology, University of Cambridge, Cambridge Biomedical Campus, Cambridge, UK; 26 NHS Blood and Transplant, Cambridge Biomedical Campus, Cambridge, UK

**Keywords:** Biomedical engineering, Computational models

## Abstract

Blood cell morphology assessment via light microscopy constitutes a cornerstone of haematological diagnostics, providing crucial insights into diverse pathological conditions. This complex task demands expert interpretation owing to subtle morphological variations, biological heterogeneity and technical imaging factors that obstruct automated approaches. Conventional machine learning methods using discriminative models struggle with domain shifts, intraclass variability and rare morphological variants, constraining their clinical utility. We introduce CytoDiffusion, a diffusion-based generative classifier that faithfully models the distribution of blood cell morphology, combining accurate classification with robust anomaly detection, resistance to distributional shifts, interpretability, data efficiency and uncertainty quantification that surpasses clinical experts. Our approach outperforms state-of-the-art discriminative models in anomaly detection (area under the curve, 0.990 versus 0.916), resistance to domain shifts (0.854 versus 0.738 accuracy) and performance in low-data regimes (0.962 versus 0.924 balanced accuracy). In particular, CytoDiffusion generates synthetic blood cell images that expert haematologists cannot distinguish from real ones (accuracy, 0.523; 95% confidence interval: [0.505, 0.542]), demonstrating good command of the underlying distribution. Furthermore, we enhance model explainability through directly interpretable counterfactual heat maps. Our comprehensive evaluation framework establishes a multidimensional benchmark for medical image analysis in haematology, ultimately enabling improved diagnostic accuracy in clinical settings.

## Abstract (web summary)

Diffusion models are reframed by developing a generative blood cell classifier that performs reliably in low-data regimes, adapts to domain shifts, detects anomalies with robustness and provides uncertainty estimates that surpass clinical expert benchmarks.

## Main

The haematological system is among the most complex physiological systems and is uniquely interconnected with all others. Though often quantified by simple ‘blood counts’ of cell class frequencies, its characteristics are both supremely rich and highly variable within and across individuals^1^. Characterizing the morphological appearances of individual blood cells as seen on light microscopy is often critical to manage haematological disorders. Complex modulation of cell morphology by diverse biological, pathological and instrumental factors requires that this task necessarily be performed by trained experts. Moreover, labelling cells by their major morphological type, for example, lymphocyte, is only the crudest form of description, on which finer fractionation into subtypes, across a wide spectrum of (ab)normality, is overlaid.

Indeed, the task of morphological characterization is both open ended and lacks a definitive ground truth: there may be morphological patterns whose subtlety has concealed great clinical importance, and some morphological classes are purely expert-determined visual phenotypes with no means of objective corroboration. Moreover, pathological appearances may be highly unusual or unique, precluding classification into any class, even at the simplest level of description, and such anomalies should be explicitly identified. The difficulty is commonly compounded by interactions with irrelevant biological features with variable representation across the population, and instrumental variations of technical origin^2,3^. The challenge, in short, is one human experts can only imperfectly meet, inevitably exhibiting marked variation with skill and experience^4,5^, and therefore, the training of machine learning (ML)-based models to automate morphological characterization is intrinsically difficult.

Recent work has applied discriminative models, particularly convolutional neural networks, to the morphological assessment of blood cells. These approaches have been used to classify leukaemia diagnosis^6^, lymphoblasts^7^, genetic acute myeloid leukaemia subtype identification^8^ and stored red blood cell morphologies^9,10^. Additionally, convolutional neural networks have been applied to the differentiation of bone marrow cell morphologies when trained on large image datasets^11^. These studies have demonstrated the potential of ML in morphological assessment, and some models have received FDA approval for clinical practice.

A desirable automated cell characterization model would have the following five key properties. First, it should be robust to domain shift and generalize to different biological, pathological and instrumental contexts and class distributions^12,13^. Second, the model should achieve high data efficiency, performing well despite sparse ground-truth labels and restricted access to comprehensive datasets as commonly found in clinical applications. Third, the decision of the model should aim to be interpretable where possible, as the reasoning behind a model’s decisions may be as important as the decisions themselves^14,15^. Fourth, the model should have the ability to identify rare or previously unseen patterns of features as such cases fall outside the model’s competence and must be so highlighted. This is particularly important in clinical applications yet often overlooked in model development and evaluation^16^. Finally, the model should be able to quantify the uncertainty attached to its decision, a feature often neglected in model assessments^17^.

Although optimally performing discriminative ML classification models can approximate human performance at classifying cells into predefined classes, they primarily learn a decision boundary based on expert labels. Consequently, they are not inherently designed to capture the full data distribution of cellular appearances. This limitation can make them less adept at handling some of the desirable properties outlined above, such as intrinsic robustness to domain shifts, natural anomaly detection for unseen cell types or high data efficiency, particularly when dealing with the complexities and variability inherent in clinical haematology data^18,19^. These open challenges limit the clinical applicability of purely discriminative approaches that only seek to replicate expert labelling.

Therefore, we introduce CytoDiffusion, a modelling approach centred around a diffusion-based generative model. Instead of merely learning a classification boundary, CytoDiffusion aims to model the full distribution of blood cell morphology. By capturing the underlying data distribution within a latent space, generative models offer several potential advantages for addressing the multifaceted challenges in clinical settings. These include facilitating greater robustness to distributional shifts, enabling inherent anomaly detection (as out-of-distribution samples are poorly represented), enhancing data efficiency, allowing interpretability through the generation of counterfactuals, and potentially streamlining the incorporation of new classes or finer subdivisions of existing ones. Classification is then performed based on this learned distributional representation, rather than being the sole objective of the model.

**Fig. 1:**
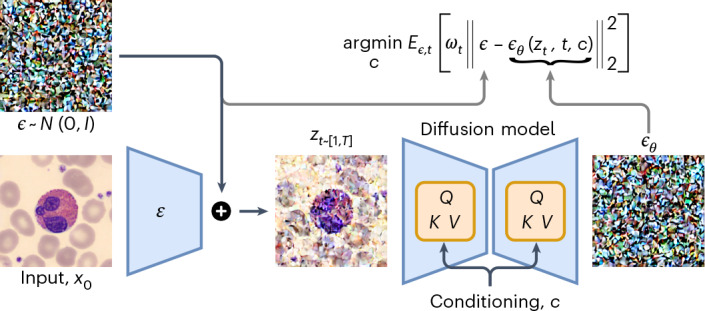
Overview of the diffusion-based classification model. Representation of the diffusion-based classification process. An input image *x*_0_ is first encoded into a latent space using an encoder ${\mathcal{E}}$. Gaussian noise ${\bf{\epsilon }} \sim {\mathcal{N}}(0,{I})$ is then added to create a noisy latent representation *z*_*t*_. This noisy representation is fed through a diffusion model for each possible class condition *c*. The model predicts the noise *ϵ*_*θ*_ for each condition. The classification decision is made by selecting the class that minimizes the error between the predicted noise *ϵ*_*θ*_ and true noise *ϵ*.

CytoDiffusion is developed and applied to real-world clinical challenges, building on recent work^20–23^ using generative models for classification challenges. We specifically chose diffusion models over alternative generative approaches based on their superior performance in modelling complex visual patterns^24^ and recent studies demonstrating their effectiveness as classifiers^20–23^. In the context of blood cell image classification, CytoDiffusion is compelled to learn the complete morphological characteristics of each cell type (by modelling the distribution) rather than focusing only on discriminative features near a decision boundary. Figure 1 illustrates the proposed modelling approach and the contributions of this work are (1) an application of latent diffusion models for blood cell image classification, (2) an evaluation framework that goes beyond accuracy and other standard metrics, incorporating domain shift robustness, anomaly detection capability and performance in low-data regimes, (3) a new dataset of blood cell images that includes artefacts and labeller confidence scores, addressing key limitations in existing datasets, (4) a principled framework for the evaluation of model and human confidence based on established psychometric modelling techniques and (5) a method for generating interpretable heat maps directly from the generative process to explain the model’s decisions.

Through these contributions, we aim to establish a standard for the development and assessment of blood cell image classification models. Our work addresses several important aspects of clinical applicability, including robustness, interpretability and reliability. We propose that the research community adopt these evaluation tasks and metrics when assessing new models for blood cell image classification. By going beyond simple discriminative statistics and other conventional benchmarks, we can develop models that are not only high performing but also trustworthy and clinically relevant.

## Results

We begin by validating the quality of images generated by CytoDiffusion through an authenticity test. Next, we assess the model’s in-domain performance on standard classification tasks across multiple datasets. We then examine CytoDiffusion’s ability to quantify uncertainty, comparing its metacognitive capabilities with those of human experts. Following this, we evaluate the model’s proficiency in anomaly detection, crucial for identifying rare or unseen cell types. We proceed to test the model’s robustness to domain shifts, simulating real-world variability in imaging conditions. Subsequently, we investigate CytoDiffusion’s efficiency in low-data regimes, a critical consideration for medical applications in which large, well-annotated datasets may be scarce. Finally, we demonstrate CytoDiffusion’s explainability through the generation of counterfactual heat maps, providing interpretable insights into its decision-making process.

### CytoDiffusion generates images indistinguishable from real images

The clinical adoption of artificial intelligence (AI) systems requires not only high performance but also trustworthiness in the model’s learned representations. To demonstrate that CytoDiffusion learns the distribution of morphological features rather than artefactual shortcuts, we conducted an authenticity test. CytoDiffusion was trained on a dataset comprising 32,619 images. To evaluate its generative performance, we enlisted ten expert haematologists to assess a total of 2,880 images (with each expert evaluating 288 images). The experts achieved an overall accuracy of 0.523 (95% confidence interval: [0.505, 0.542]) in distinguishing between real and synthetic images, with a sensitivity of 0.558 and a specificity of 0.489. This performance is comparable to random guessing, indicating that the synthetic images produced by CytoDiffusion are almost indistinguishable from real blood cell images, even to experienced professionals.

In addition, the quality of conditional synthesis was evaluated by comparing the experts’ cell type classifications of the synthetic images with the conditioning labels used during generation. The high agreement rate of 0.986 not only validates the quality of generation but also confirms that CytoDiffusion preserves class-defining morphological features. The ability to generate synthetic images indistinguishable from real ones indicates that CytoDiffusion has learnt the morphological distribution of blood cell appearances. Examples of generated images are shown in Supplementary Fig. 2.

### CytoDiffusion demonstrates competitive classification performance

**Extended Data Fig. 1:**
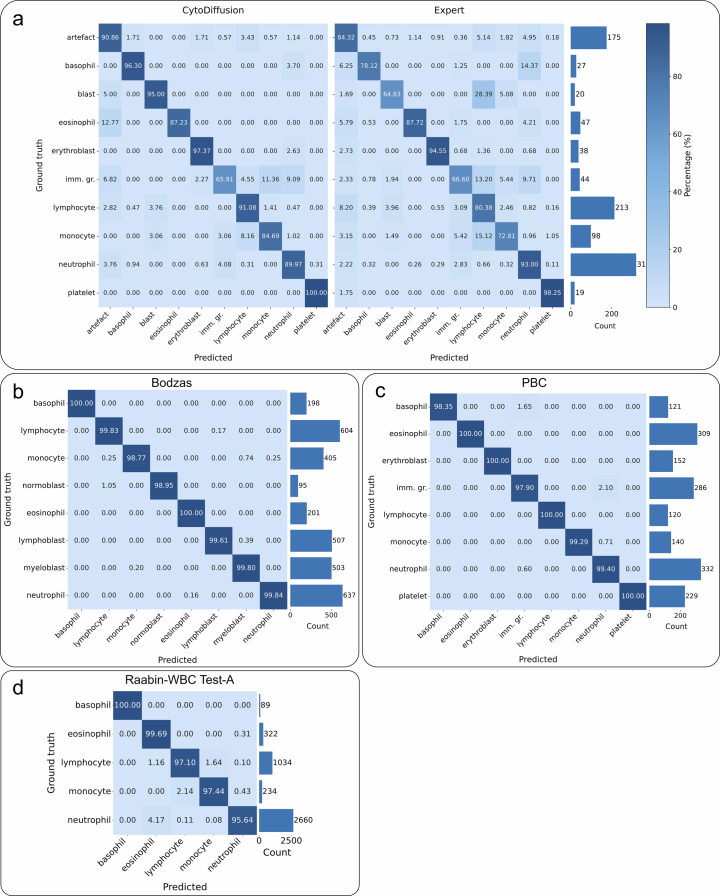
Classification confusion matrices. Confusion matrices showing CytoDiffusion’s classification performance across datasets. (**a**) CytoData comparison: left matrix shows CytoDiffusion results, right shows average human expert performance (where each expert was evaluated against a consensus ground truth derived from all other experts). CytoDiffusion’s performance on Bodzas (**b**), PBC (**c**), Raabin-WBC Test-A (**d**).

Although the primary focus of our work lies in uncertainty quantification, anomaly detection, robustness to domain shifts, efficiency in low-data scenarios and explainability, we first establish CytoDiffusion’s baseline performance on standard classification tasks to ensure that its foundation is robust. We evaluated CytoDiffusion across four datasets: CytoData (our custom dataset), Raabin-WBC^25^, PBC^26^ and Bodzas^27^. As shown in Table 1, CytoDiffusion achieves state-of-the-art performance on CytoData, PBC and Bodzas, demonstrating that our diffusion-based approach can match or exceed the capabilities of traditional discriminative models. Extended Data Fig. 1 shows the classification confusion matrices for CytoDiffusion across all four datasets.

**Table 1:** Comparison of model performance across four datasets

Dataset	Method	Year	Accuracy	F1	Sensitivity	Precision
CytoData	ViT-B/16	2024	0.8440	0.8166	0.8110	$\underline{0.8502}$
	EfficientNetV2-M	2024	$\underline{0.8790}$	$\underline{0.8512}$	$\underline{0.8533}$	**0.8613**
	CytoDiffusion (this work)	2024	**0.8940**	**0.8690**	**0.8984**	0.8500
Raabin-WBC Test-A	Ref. ^52^	2021	0.9465	0.9104	0.9276	0.8979
	Ref. ^25^	2022	**0.9917**	**0.9845**	0.9795	**0.9903**
	Ref. ^53^	2022	0.9871	$\underline{0.9778}$	**0.9842**	0.9718
	Ref. ^54^	2022	0.9517	0.9171	0.9340	0.9343
	Ref. ^55^	2023	0.9829	0.9708	0.9769	–
	Ref. ^50^	2023	$\underline{0.9875}$	0.9636	0.9739	0.9560
	Ref. ^49^	2024	0.9780	0.9769	$\underline{0.9817}$	0.9726
	ViT-B/16	2024	0.9620	0.9422	0.9752	0.9184
	EfficientNetV2-M	2024	0.9871	0.9772	0.9803	$\underline{0.9742}$
	CytoDiffusion (this work)	2024	0.9647	0.9489	0.9797	0.9274
PBC	Ref. ^26^	2019	0.9616	0.9615	0.9616	0.9622
	Ref. ^56^	2020	0.9763	0.9760	0.9761	0.9659
	Ref. ^57^	2022	0.9202	0.9093	0.9180	0.9036
	Ref. ^38^	2023	0.9300	0.9000	0.9000	0.9100
	Ref. ^58^	2023	0.9725	0.9727	0.9725	0.9730
	Ref. ^37^	2024	0.9889	0.9891	0.9891	0.9893
	ViT-B/16	2024	0.9819	0.9817	0.9845	0.9793
	EfficientNetV2-M	2024	$\underline{0.9913}$	$\underline{0.9912}$	$\underline{0.9914}$	$\underline{0.9911}$
	CytoDiffusion (this work)	2024	**0.9935**	**0.9946**	**0.9937**	**0.9956**
Bodzas	ViT-B/16	2024	0.9889	0.9898	0.9880	0.9916
	EfficientNetV2-M	2024	$\underline{0.9943}$	$\underline{0.9943}$	$\underline{0.9942}$	$\underline{0.9944}$
	CytoDiffusion (this work)	2024	**0.9965**	**0.9965**	**0.9960**	**0.9970**

F1, sensitivity and precision values are reported as macro averages^59–78^.

Bold values indicate the best result for each metric within a given dataset.

${{\rm{Underlined}}}$ values indicate the second-best result.

### CytoDiffusion outperforms human experts in uncertainty quantification

The biological realm is characterized by constitutional, incompletely reducible uncertainty. In every task, it is valuable to quantify not only the fidelity but also the uncertainty of the agent: human or machine. Metacognitive measures of this kind enable the qualification of predictions, stratification of case difficulty and principled ensembling of agents^28,29^.

Our dataset uniquely incorporates human expert confidence for all images, providing a rare opportunity to compare model uncertainty with human expert uncertainty. Quantifying uncertainty is complicated by two cardinal aspects of the task. First, uncertainty has no reliable ground truth in real-world settings. Second, uncertainty in the present context contains an aleatoric component (the constitutional discriminability of the classes) and an epistemic component (the agent’s ability to discriminate between them). The former is determined by the domain and the latter, by the characteristics of the agent. In the ideal case, the epistemic uncertainty is zero, and the agent’s uncertainty is wholly aleatoric, determined by how much discriminant signal the data contains. In such a case, the relation between uncertainty and accuracy ought to approximate that of an ideal psychophysical observer detecting a noisy signal, that is, the uncertainty measure should resemble discriminability.

**Fig. 2:**
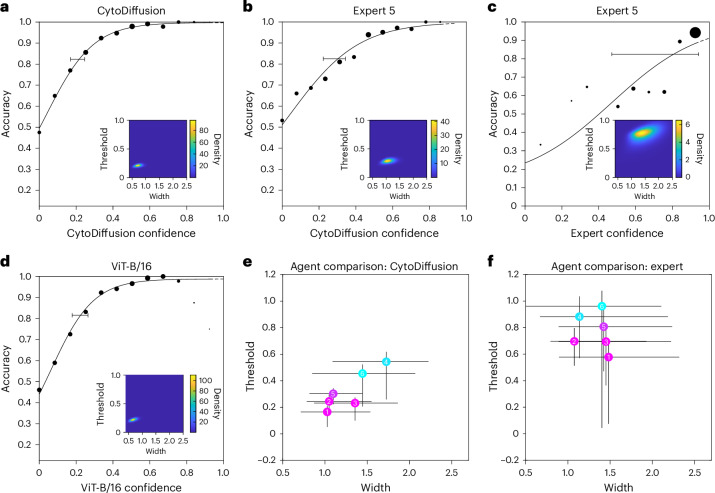
Bayesian psychometric analysis of model and expert performance. Performance was evaluated on our custom CytoData test set (*n* = 1,000 images). **a**–**d**, Psychometric functions showing accuracy as a function of a discriminability index. In these panels, data points (black circles) represent the mean accuracy for images binned by confidence, and their size is proportional to the number of trials in each bin. The solid black line is the maximum-likelihood psychometric function fit to the data. The horizontal black error bar on the curve indicates the 95% credibility interval for the function’s threshold, estimated at 80% accuracy (unscaled by lapse and guess rates). The plots in the inset show the joint posterior probability density for the psychometric function’s parameters, width and threshold. **a**, Psychometric function for CytoDiffusion, with its own confidence score as the discriminability index. **b**, Psychometric function for a representative human expert (Expert 5), using CytoDiffusion’s confidence score as the discriminability index. **c**, Psychometric function for the same expert (Expert 5), using expert confidence as the discriminability index. **d**, Psychometric function for the ViT-B/16 model, with its own confidence score as the discriminability index. **e**,**f**, Comparison of psychometric function parameters (width and threshold) across the six human experts. The coloured circles represent the posterior mean of the parameter estimates, and the error bars represent the 95% credibility intervals. Parameters were estimated using either CytoDiffusion confidence (**e**) or mean expert confidence (**f**) as the index of signal strength. Source data

This insight allows us to deploy the mature conceptual apparatus of psychometric function estimation to the task of evaluating an agent’s uncertainty. We use well-established Bayesian psychometric modelling techniques to derive a psychometric function for CytoDiffusion’s performance (Fig. 2a), revealing an excellent fit, with tight posterior distributions on the key threshold and width parameters (Fig. 2a, axes in the inset). Although direct measurement is impossible, this suggests CytoDiffusion’s uncertainty is dominated by the aleatoric component and its behaviour is close to that of an ideal observer.

This conclusion is reinforced by evaluating individual human expert performance, judged against expert consensus, with CytoDiffusion’s confidence as the measure of discriminability. The resultant function, illustrated for Expert 5 (Fig. 2b), not only exhibits a good fit but also describes the relationship better than consensus human expert confidence (Fig. 2c), suggesting that CytoDiffusion’s metacognitive abilities are superior to human experts here. We also applied the same psychometric analysis to the vision transformer (ViT)-B/16 model (Fig. 2d). Although the psychometric function shows a good fit, the data points for ViT-B/16 exhibit non-monotonic behaviour at higher confidence values. This suggests that the discriminative model’s confidence estimates are less reliable precisely when high certainty would be most clinically valuable, unlike CytoDiffusion that maintains a consistent relationship between confidence and accuracy. Examination of the estimated threshold and width parameters for each human expert, with CytoDiffusion (Fig. 2e) or human expert (Fig. 2f) confidence, shows that CytoDiffusion’s measure can distinguish between the varying abilities of human experts better than they themselves can.

### CytoDiffusion excels at detecting anomalous cell types

**Fig. 3:**
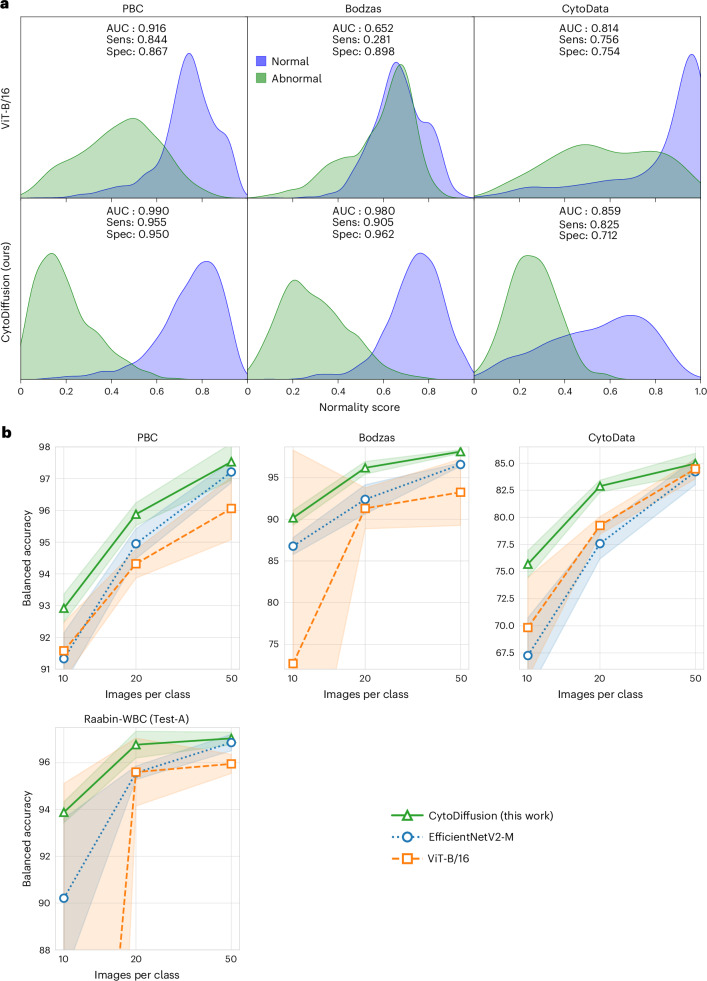
Anomaly detection and low-data performance comparison. **a**, Kernel density estimate figures comparing the anomaly detection performance of ViT-B/16 (top row) with CytoDiffusion (bottom row) for erythroblasts (left and right columns) and blasts (middle column). The horizontal axis represents the normality score, normalized to [0, 1]. The sensitivity (Sens) and specificity (Spec) values show each model’s performance in detecting anomalous cells and correctly classifying normal samples. **b**, Model performance comparison under low-data conditions across four cytology datasets. The data points represent the mean balanced accuracy, and the shaded areas represent the standard deviation. Statistics were calculated from five independent training sessions. AUC, area under the curve. Source data

For each dataset, we evaluate our model’s performance in detecting clinically important anomalous cell types. The detection of blast cells is crucial in screening for various haematological malignancies, particularly leukaemia and myelodysplastic syndromes, where high sensitivity is essential to minimize false negatives that could lead to missed diagnoses. As shown in Fig. 3a, for the Bodzas dataset, with blasts as the abnormal class, CytoDiffusion achieved both high sensitivity (0.905) and specificity (0.962). However, the ViT suffered from extremely poor sensitivity (0.281), making it inadequate for clinical applications.

For PBC and CytoData, both with erythroblasts as abnormal, CytoDiffusion achieved a higher sensitivity compared with ViT and maintained a high specificity. These results demonstrate our model’s ability to distinguish between normal cells it was trained on and abnormal cell types not present in the training data, as well as maintaining the high sensitivity required for clinical applications.

### CytoDiffusion shows robustness to domain shifts

To assess generalizability, we evaluated models across datasets with varying domain shifts. Models trained on Raabin-WBC were tested on Test-B (different microscopes and cameras) and LISC (different microscopes, cameras and staining). Models trained on CytoData were tested on PBC and Bodzas, where the PBC dataset was created using a different generation of CellaVision technology (DM9600 for CytoData and DM96 for PBC). The Bodzas dataset introduces another domain shift as it was manually stained, rather than using automated staining procedures. As shown in Extended Data Table 1, CytoDiffusion achieves state-of-the-art accuracy on all four datasets. These consistent performance advantages across varying degrees of domain shift demonstrate CytoDiffusion’s robustness to dataset variations, suggesting good generalization capabilities for real-world clinical applications.

### CytoDiffusion outperforms discriminative models in low-data scenarios

To evaluate performance under limited-data conditions, we conducted experiments across the four previously described cytology datasets. For each dataset, we conducted training with limited subsets of 10, 20 and 50 images per class, simulating conditions of sparse data availability. Figure 3b demonstrates that CytoDiffusion consistently outperforms the discriminative models EfficientNetV2-M and ViT-B/16 across all four datasets. The advantage is particularly pronounced in the most data-scarce conditions, where traditional discriminative approaches struggle to generalize effectively.

### CytoDiffusion provides visual explanations through counterfactual heat maps

**Fig. 4:**
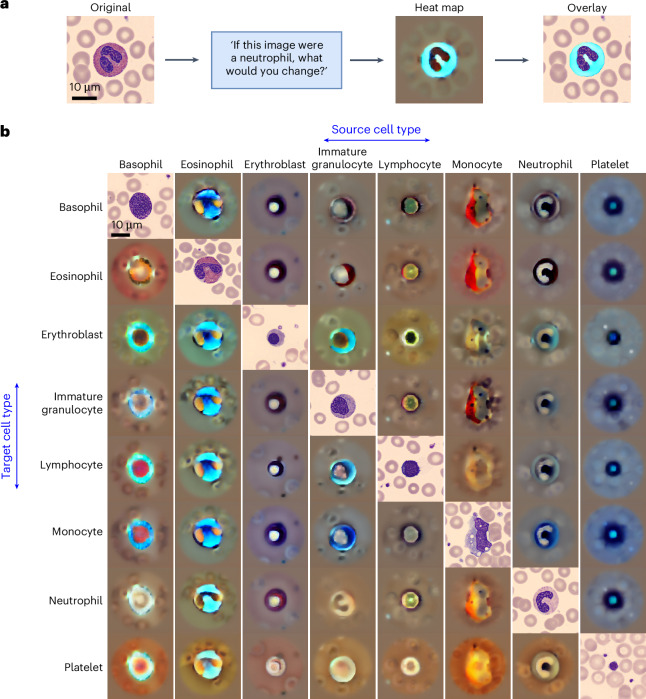
Counterfactual visualizations for model explainability. **a**, An example of generating a counterfactual explanation. Left: original image of an eosinophil. Centre right: counterfactual heat map (**H**_neutrophil_), which highlights areas that would need to change for the model to classify the image as a neutrophil. Far right: an overlay of the thresholded heat map on the original image, localizing the most critical features. **b**, Matrix of counterfactual heat maps for various cell-type transitions. The diagonal displays original images of each cell type, which serve as the source image for their respective columns. Each off-diagonal element in the same column represents a counterfactual heat map (**H**_*c*_) showing the transition from the diagonal element (source) to the cell type of that row (target). Areas in the heat map with colours that deviate most from the background indicate regions in which there are large errors in the latent space between the two classes.

Counterfactual heat maps highlight the regions of an image that would need to change for it to be classified as a different cell type. In Fig. 4a, we used an eosinophil as an example and prompted the model to consider what alterations would be necessary for this cell to be classified as a neutrophil, generating a heat map (**H**_neutrophil_) that highlights regions in which there are large errors in the latent space between the two classes. The overlay of this heat map on the original image reveals that the model focuses primarily on distinguishing granularity between neutrophils and eosinophils, with areas of large colour deviation from the background indicating the most critical regions of difference.

To provide a comprehensive view of CytoDiffusion’s abilities across all cell types, Fig. 4b shows the generated counterfactual heat maps for each possible class transition in the PBC dataset. This visualization offers insights into the model’s decision-making process for each cell type. For instance, when considering the transition from neutrophil to eosinophil (row 2, column 7), the model highlights regions in the cytoplasm (darker areas) where features should be added, largely maintaining the nuclear shape.

In particular, the heat maps also reveal the model’s understanding of subtle differences between similar cell types. In the transition from monocyte to immature granulocyte (Fig. 4b, row 4, column 6), the model indicates the difference in cytoplasm between the more acidophilic cytoplasm of the immature granulocytes compared with the greyish-blue monocytic cytoplasm. Intriguingly, the model also suggests the filling of the monocytic vacuoles (appearing as dark spots in the heat map). This captures one of the typical morphological findings in monocytes differentiating them from other normal blood cells and demonstrates the model’s ability to focus on nuanced information. These visualizations also serve as a validation tool, enabling the identification of potential model biases by revealing whether the model is focusing on clinically irrelevant areas during classification. This transparency in the decision-making process makes the model more trustworthy for clinical applications, as practitioners can verify that classifications are based on legitimate morphological features rather than artefacts or spurious correlations.

## Discussion

This study introduces not only CytoDiffusion for haematological cell image classification but also a principled evaluative framework. Our approach is motivated by the desire to achieve a model with superhuman fidelity, flexibility and metacognitive awareness that can capture the distribution of all possible morphological appearances. These ambitions are attainable only by eliciting structure in the data beyond that which can be obtained simply from the expert labelling of images. Both our methodology and evaluative framework are grounded in the recognition of the inherent complexity of the target system along with the inherent constraints for AI modelling with medical imaging data, for example, relatively small dataset regimes and instrumental variation reflected in images. In addition, we propose that a comprehensive evaluation of medical imaging models should include multiple complementary assessment criteria. Although standard performance metrics are essential, we have deliberately evaluated our approach across several key dimensions: (1) robustness to domain shift, (2) ability to detect anomalies, (3) effectiveness with limited training data, (4) reliability in uncertainty quantification and (5) interpretability of outputs. By assessing all these aspects within a single study, we aim to provide a more complete picture of model capabilities and limitations relevant to clinical deployment.

Robustness of a classification model to domain shift, that is, its ability to generalize across different imaging conditions, is crucial for its practical application in clinical settings. This generalization capability is particularly important in haematology, where variations in microscope types, camera systems and staining techniques are common across different laboratories and hospitals. A model that performs well only under specific imaging conditions would have limited utility in real-world clinical practice, potentially leading to inconsistent or unreliable diagnoses when deployed in new environments. Additionally, the ability to identify rare or unexpected cell types is crucial in clinical scenarios, where the detection of abnormal cells can have important diagnostic implications. Furthermore, our assessment of model performance with reduced training examples examines the relationship between data availability and classification efficacy, illustrating the learning efficiency of the system. This feature is particularly beneficial for effectively handling rare or minority cell types, thereby influencing model selection and deployment decisions. Such data efficiency will be crucial when dividing classes into more granular subclasses encountered in clinical haematological assessments, many of which may only be sparsely represented.

Additionally, in any classification task, whether performed by a human or an ML algorithm, it is highly informative to understand the uncertainty in the final decision, a surrogate for how difficult the sample is to classify. This is particularly true for clinical data in which the classification result can inform an intervention or treatment decision. Currently, a method for the optimal evaluation of model uncertainty is not established in the ML literature. We introduce a framework for evaluating an agent’s uncertainty—human or machine—based on the expected structure of the purely aleatoric uncertainty of an ideal psychophysical observer. This approach allows us to quantify model uncertainty as departure from the ideal psychometric function relating purely aleatoric uncertainty to fidelity. We demonstrate that CytoDiffusion produces superior uncertainty estimates compared with human experts, with two key clinical implications. First, our approach enables efficient triage: cases with high certainty can be processed automatically, whereas uncertain cases can be flagged for human review. Second, the transparent quantification of model uncertainty may help build essential trust amongst clinical practitioners. Additionally, it provides a principled mechanism for weighting ensembles of models based on their confidence and for detecting domain shifts or equipment malfunctions through changes in the uncertainty distribution.

Moreover, interpretability remains essential for the clinical deployment of ML models. Although discriminative approaches can generate post hoc explanations through techniques like Grad-CAM or LIME^30,31^, CytoDiffusion generates counterfactual heat maps as direct outputs of the model’s generative process. These heat maps highlight regions that would need to change for an image to be classified differently, providing immediate insights into the morphological distinctions the model identifies between cell types. A key advantage of our approach is that these visualizations emerge naturally from the model’s operation without requiring additional interpretive layers, preserving the integrity of the decision-making rationale.

A key strength of our generative approach is its ability to learn a comprehensive representation of the data distribution, a capability that underpins its strong performance and holds potential for future discovery. This deep representational learning is the plausible mechanism for the model’s demonstrated success in tasks such as anomaly detection and robustness to domain shift. Effectively identifying an unseen cell type as anomalous relies on the model having learned a high-fidelity distribution of normal morphologies, going beyond the minimal features needed for simple classification. This is crucial because morphologically defined cell types are not natural but rather phenotypes defined by visual distinguishability combined with clinical utility. Crucially, some functionally distinct cell types may be visually indistinguishable, whereas others differ markedly in morphology despite functional relatedness. Thus, physiological or pathological relevance cannot be constrained to human intuition alone. Although the current study demonstrates the existence of this rich representational space, the explicit exploration of this space to identify novel, clinically important subclasses remains a promising direction for future work. For example, the learned representations could be used to characterize heterogeneities within existing classes, facilitating the identification of new morphological signals that can subsequently be evaluated for clinical relevance.

Our results suggest that CytoDiffusion can build on existing discriminative approaches by addressing key challenges in clinical deployment, from domain shift and uncertainty quantification to interpretable visual explanations and data efficiency, and maintaining competitive performance on standard metrics, without requiring extensive hyperparameter tuning or data-specific architectural modifications.

Our approach is not without limitations. The inference process of CytoDiffusion is computationally expensive, scaling with the number of classes. However, this is less problematic in the medical domain, where datasets typically have far fewer classes than general image classification tasks like ImageNet^32^. Moreover, CytoDiffusion’s adaptive allocation of computing resources, dedicating more effort to challenging images, makes it more effective. For future applications with more granular division of blood cell types (for example, subdividing blasts into myeloblasts, lymphoblasts and monoblasts), recent work^33^ on hierarchical diffusion classifiers suggests a promising direction for maintaining computational efficiency through progressive category pruning. CytoDiffusion required an average of 42 iterations per image (0.043 seconds per iteration), resulting in a mean classification time of 1.8 seconds per image. Several optimizations could further improve efficiency: code optimization and model distillation should reduce computational requirements, parallelization across multiple computing resources would enable the simultaneous processing of images and advancing hardware capabilities will naturally decrease relative computational costs over time.

There are many possible extensions to this study. First, we have not fully exploited the representation learning capabilities of generative models, where there is potential for further advances, for example, through the use of generative modelling architectures with compact latents. Moreover, generative models enable the detection and assurance of equity in medical diagnostics. Although not implemented in this study, conditioning on minority characteristics would allow for legibility of differences in the model’s perception across diverse demographic groups^34^, and potentially enable augmentation with counterfactually transformed images to mitigate the impact of class imbalance on equity. This capability is crucial for both detecting potential biases and enabling targeted remedial actions, ensuring fair and equitable application of AI in healthcare.

In conclusion, our generative approach with CytoDiffusion, combined with a comprehensive evaluation framework, offers a promising step towards more robust, interpretable and trustworthy AI systems in healthcare. Future work should not only refine these methods and assess their applicability to other medical imaging domains but also explicitly test their ability to promote fairness and mitigate bias.

## Methods

### Diffusion classifiers

Recent studies have shown that diffusion models can also be utilized as classifiers^20–23^. We use a latent diffusion model (Supplementary Section 1). Given an input image **x**, we want to predict the most probable class $\hat{c}$. This can be formalized as finding the class $\hat{c}$ that maximizes the posterior probability *p*(*c* = *c*_*k*_∣**x**). Using Bayes’ theorem, this is equivalent to

1 $$\begin{array}{l}\hat{c}=\mathop{{\rm{argmax}}}\limits_{{c}_{k}}\,p(c={c}_{k}| {\bf{x}})=\mathop{{\rm{argmax}}}\limits_{{c}_{k}}\,p({\bf{x}}| c={c}_{k})\cdot p(c={c}_{k})\\\quad=\mathop{{\rm{argmax}}}\limits_{{c}_{k}}\log p({\bf{x}}| c={c}_{k})\,,\end{array}$$

assuming a uniform prior over the classes, $p(c={c}_{k})=\frac{1}{K}$, where *K* is the number of classes.

As we do not have direct access to the (negative) log likelihood, we use our loss function to approximate it instead and, therefore, take

2 $$\hat{c}=\mathop{{\rm{argmin}}}\limits_{c}{{\mathbb{E}}}_{{\bf{\epsilon }},t}\left[{w}_{t}{\left\Vert {\bf{\epsilon }}-{{\bf{\epsilon }}}_{\theta }({{z}}_{t},t,c)\right\Vert }_{2}^{2}\right],$$

with the weight *w*_*t*_ discussed in Supplementary Section 1. To achieve this, we randomly pick a time step *t* and noise *ϵ* and calculate the error as

3 $${\left\Vert {\bf{\epsilon }}-{{\bf{\epsilon }}}_{\theta }({{z}}_{t},t,c)\right\Vert }_{2}^{2}\,,$$

for all class labels *c*. These error values are then normalized using *w*_*t*_ and the results are stored.

The process then repeats with newly sampled values of *t* and *ϵ*. Supplementary Fig. 4 provides an intuitive explanation of how our model makes its predictions.

Following the methodology in ref. ^22^, we repeatedly gather new sets of errors for each candidate class. Classes that are less likely to have the lowest error are progressively eliminated. This process can be interpreted as a successive elimination algorithm for best-arm identification in multi-armed bandit settings^35,36^. The elimination is achieved using a paired Student’s *t*-test.

Given that the errors do not perfectly follow the standard assumptions of a Student’s *t*-test, we use the same safeguards as those in ref. ^22^: a conservative *P* value of 2 × 10^−3^ and a requirement that each class must be scored a minimum of 20 times before elimination to minimize the chance of incorrectly pruning the correct class. This iterative procedure continues until there is only one class left or a maximum of 2,000 iterations is reached. Supplementary Fig. 1 provides an analysis of different *P* values, as well as various minimum and maximum numbers of iterations.

### General training setup

Unless otherwise specified, we used the following training configuration for all experiments. We used Stable Diffusion 1.5 (ref. ^24^) as our base model. For class conditioning, we bypassed the tokenizer and text encoder, directly feeding the model with one-hot-encoded vectors for each class, replicated vertically and padded horizontally to match the expected matrix of 77 × 768 dimensions. We utilized a batch size of 10, a learning rate of 10^−5^ with linear warm-up over 1,000 steps and trained on an A100-80GB GPU. Details of the training and inference parameters are provided in Supplementary Section 2.

### Datasets

We utilized multiple datasets (described in Extended Data Table 2 and Supplementary Table 2), including four that are publicly available and one custom dataset, CytoData, to develop and evaluate our diffusion classifier for haematological cell image classification. CytoData, available at https://www.ebi.ac.uk/biostudies/studies/S-BSST2156, is an anonymized dataset consisting of 559,808 single-cell images from 2,904 blood smear slides obtained from Addenbrooke’s Hospital in Cambridge, UK, with a labelled subset of 4,996 images across ten classes. These images were created using CellaVision DM9600, a specialized imaging technology for cellular analysis. The labelling strategy is described in Supplementary Section 3. In particular, when labelling CytoData, we included an artefact class, addressing a critical challenge in clinical applications, as blood smear slides often contain artefacts that may be mistaken for cells by deep-learning-based cell detection models. By explicitly modelling these artefacts, CytoData aims to enhance clinical applicability. Furthermore, a distinctive feature of CytoData is the inclusion of labeller confidence scores, which provides valuable information for analyses beyond simple correlations.

### Authenticity test

To assess the quality and authenticity of our fine-tuned diffusion model’s synthetic blood cell images, we conducted an authenticity test with expert haematologists. This evaluation was designed to determine whether the model could effectively capture the underlying distribution of blood cell images across various cell types, a capability that traditional discriminative models are not inherently required to possess. Additionally, we sought to assess the accuracy of the generated cell types. Further details are provided in Supplementary Section 4. Ten haematology specialists from our research group, with {34, 28, 25, 15, 10, 9, 6, 5, 5, 1} years of experience in blood microscopy, participated in the authenticity test. Participants were informed that half of the images presented to them would be real images from our dataset, whereas the other half would be synthetic images generated by our model. Each specialist was presented with the 288 images in a randomized order and asked to perform two tasks: (1) identify whether each image was synthetic or real and (2) classify each image into one of the nine designated blood cell types.

### In-domain performance

To establish a baseline for CytoDiffusion, we evaluated its performance on standard in-domain classification tasks using four datasets: CytoData, Raabin-WBC, PBC and Bodzas. Although we note that these datasets use different train–validation–test proportions, we have deliberately maintained these differences to ensure consistency with established benchmarks in the literature. For the Raabin-WBC dataset, we used the predefined test set (Test-A) provided by the dataset authors, and allocated 10% of the provided training data for validation. For the PBC dataset, following prior research, we used an 80–10–10 split for train–validation–test^37,38^. For both CytoData and Bodzas, we implemented a 70–10–20 split. Additionally, for the Bodzas dataset, in accordance with refs. ^39,40^, we merged the neutrophil class.

For all the datasets, we trained our model for 72,000 steps. To provide a basis for comparison, we also trained and evaluated EfficientNetV2-M and ViT-B/16 models under similar conditions. It is important to note that we have excluded some studies from our comparison due to methodological differences that could lead to unfair or misleading comparisons. Specifically, we are not comparing with papers that do not have a conventional train–validation–test split^41,42^ or those that do not test on the predefined test set^37,43^.

### Uncertainty measure

To evaluate the quality of CytoDiffusion’s uncertainty measure, we exploited the decomposability of uncertainty into model and aleatoric components. An ideal model—indeed any ideal agent—should contribute no uncertainty of its own, leaving aleatoric uncertainty as the sole residue. If so, the uncertainty measure should reduce to the magnitude of the discriminative signal, and the relation between the uncertainty measure and model fidelity should conform to that of an ideal observer of a noisy signal. This allows us to exploit psychometric function modelling to quantify how close an agent’s uncertainty—machine or human—is to the aleatoric floor^44^. Our analysis used CytoDiffusion and ViT-B/16 models, both trained on the CytoData outlined in the ‘In-domain performance’ section. For quantifying the model’s uncertainty, we calculated the difference between the two classes with the smallest error, rescaled in the interval [0, 1]. For the ViT model, uncertainty was quantified as the difference between the two classes with the largest pre-activation values (logits), also rescaled to the interval [0, 1]. To establish a measure of labeller uncertainty, we mapped the confidence levels provided by our expert haematologists to numerical values: 1.0 for high confidence, 2/3 for moderate confidence, 1/3 for low confidence and 0 for no confidence. For each image, we then calculated the mean confidence score across all experts who labelled that image, providing a single aggregate measure of expert confidence per image.

Psychometric functions describe the relation between the performance of an observer and a (typically scalar) property of the observed^45^. The performance of interest is usually detection or classification, expressed as a function of signal strength on a monotonically increasing scale. Since an ideal model is as confident as the data allows, exhibiting purely aleatoric uncertainty, we can quantify the proximity of a model to that ideal by fitting a psychometric function with model confidence as the index of signal strength. A good measure of uncertainty should conform closely to an ideal observer, yielding a sigmoid curve rising from a chance guess rate *γ*, where uncertainty is the maximum, to a lapse rate *λ*, where the uncertainty is minimum and any errors are not explicable by insufficient information (Supplementary Section 5). For *γ*, we fix the parameter at 1/10, reflecting the ten possible classes, and for *λ*, we use a beta distribution with parameters (1, 10). We report the threshold and width estimated parameters, and their posterior distributions, citing 95% credibility intervals.

### Anomaly detection

To evaluate our model’s capability for detecting anomalous cell types, we designed an experiment that simulates real-world scenarios in which rare or previously unseen cell types might appear in clinical samples. This approach involved excluding specific abnormal cell classes during training and assessing the model’s ability to identify these classes during testing. We utilized three datasets for this experiment: Bodzas, PBC and CytoData. For the Bodzas dataset, we excluded the blast class, which comprised both lymphoblasts and myeloblasts (5,036 images in total). From both PBC and CytoData datasets, we excluded the erythroblast class (1,513 and 191 images, respectively). We use a normality score inspired by other work^46,47^ to quantify the model’s confidence (Supplementary Section 6). To visualize each model’s ability to distinguish between normal and abnormal cells, we generated kernel density estimation curves of the normality score for both groups. To quantify this ability, we calculated sensitivity, specificity and area under the curve for each model and dataset.

### Domain shift

We assessed robustness under domain-shift conditions using multiple datasets. For the Raabin-WBC^25^ and LISC^48^ datasets, we followed the methodologies outlined in previous studies^49,50^. Specifically, we utilized Raabin-WBC’s predefined train split (90% training and 10% validation) and evaluated on its Test-B split. The Test-B split was created using a different microscope and camera type compared with the training and validation sets, introducing a domain shift. Additionally, we used the LISC dataset as a second test set, which was created using different microscope and camera types, as well as a different staining method, further increasing the domain-shift challenge. To maintain consistency with previous studies^49,50^ and accommodate the lower resolution of LISC images, we resized all images to 224 × 224 pixel^2^. We used a batch size of 32 and trained for 22,000 steps on an NVIDIA RTX A5000 GPU. For comparison, we also fine-tuned and tested EfficientNetV2-M and ViT-B/16.

Additionally, we evaluated our models’ robustness to domain shift by training the models on CytoData and applying them to the PBC and Bodzas datasets. However, since the Bodzas dataset was created using a different zoom compared with CytoData, we applied zoom augmentation (random zoom factor uniformly selected between 1.0 and 2.2) during training. The training and evaluation processes were repeated five times for each model to ensure reliable performance estimates.

### Efficiency in low-data regimes

To evaluate the performance of our model when limited training data are available, we conducted experiments across four datasets: CytoData, Raabin-WBC, PBC and Bodzas. For each dataset, we created low-data environments by randomly sampling 10, 20 or 50 images per class from the training sets. For these low-data subsets, we trained CytoDiffusion for 30,000, 50,000 and 150,000 steps, respectively, and saving checkpoints every 1,500, 5,000 and 10,000 steps. For each subset, we selected the checkpoint with the highest validation accuracy for testing. For comparison, we also trained and evaluated EfficientNetV2-M and ViT-B/16 models. To account for variability, we repeated the entire experiment five times, each time randomly resampling new image sets at each of the three data volumes that were then used consistently across all three model architectures. All models were trained on an NVIDIA RTX A5000 GPU.

### Explainability

One of the integral aspects of CytoDiffusion’s utility in clinical settings is its ability to provide explainable predictions. To achieve this, we use a counterfactual heat-map approach, which elucidates what changes would be necessary for an image to be classified under a different specified class. This method is particularly beneficial for understanding model decisions in complex medical imaging tasks such as the classification of blood cell types. Initially, we calculate the difference between the original noise *ϵ* and the noise predicted by the model for each class condition *c*. This difference is recorded for all iterations, and the mean error for each condition *c* is computed as ${\varDelta }_{c}=\frac{1}{N}\mathop{\sum }\nolimits_{n = 1}^{N}\left({{\bf{\epsilon }}}_{n}-{{\bf{\epsilon }}}_{\theta }({{z}}_{{t}_{n}},{t}_{n},c)\right)\,$, where *N* is the number of iterations. Subsequently, for each class condition *c*, we calculate the deviation from the condition with the minimum error, designated as ${\varDelta }_{\hat{c}}$, where $\hat{c}$ is the predicted class. The adjusted *δ*_*c*_ is then ${\delta }_{c}={\varDelta }_{c}-{\varDelta }_{\hat{c}}\,$. Finally, the *δ*_*c*_ values are decoded back to the pixel space using the variational autoencoder decoder to obtain the counterfactual heat maps ${{\bf{H}}}_{c}={\mathcal{D}}({\delta }_{c})\,$, where ${\mathcal{D}}$ denotes the variational autoencoder decoder and **H**_*c*_ represents the heat map for condition *c*. These heat maps visually represent modifications that would shift the image classification from the predicted class to the target class *c*, thereby providing a powerful tool for explaining and validating model predictions.

### Reporting summary

Further information on research design is available in the Nature Portfolio Reporting Summary linked to this article.

## Supplementary information

Supplementary Information Supplementary Sections 1–11, Figs. 1–5, Tables 1 and 2 and details.

Reporting Summary

## Source data

Source Data Fig. 2 Source data for Fig. 2.

Source Data Fig. 3 Source data for Fig. 3.

## Figures and Tables

**Extended Data Table 1:**
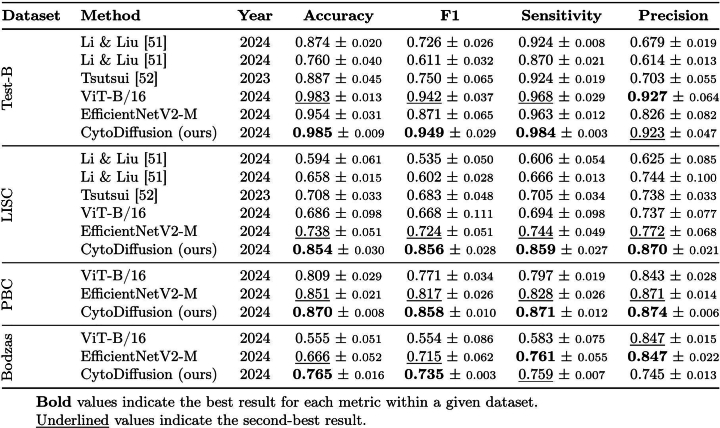
Model performance under domain shift

Performance under domain shift conditions. Models were trained on Raabin-WBC (for Test-B and LISC evaluations) or CytoData (for PBC and Bodzas evaluations). All scores are reported as mean ± standard deviation from five independent training runs.

**Extended Data Table 2:**
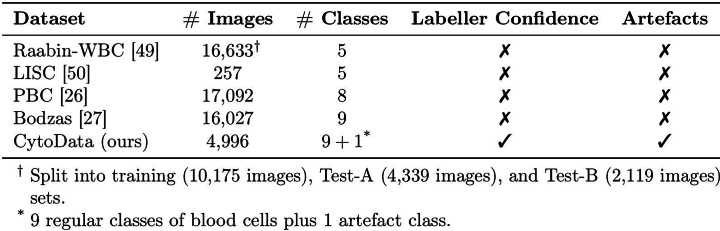
Summary of study datasets

Summary of the datasets used in our experiments.

## Data Availability

CytoData is available at https://www.ebi.ac.uk/biostudies/studies/S-BSST2156 and was obtained under an approved study protocol (IRAS 303792). The other datasets used in this analysis are publicly available as referenced: Raabin-WBC^25^, LISC^48^, PBC^26^ and Bodzas^27^. Source data are provided with this paper.

## Code availability

All code is available via GitHub at https://github.com/CambridgeCIA/CytoDiffusion and via Zenodo at 10.5281/zenodo.14825813 (ref. ^51^).
