# Supplementary material for: Deep generative classification of blood cell morphology
Source: Nat Mach Intell. 2025 Nov 19;7(11):1791–803. doi: 10.1038/s42256-025-01122-7 (PMC12629977; doi:10.1038/s42256-025-01122-7)
Supplement: Supplementary file 1 — Supplementary Sections 1–11, Figs. 1–5, Tables 1 and 2 and details. [file 42256_2025_1122_MOESM1_ESM.pdf]

nature machine intelligence

Supplementary information https://doi.org/10.1038/s42256-025-01122-7

Deep generative classification of blood cell morphology

In the format provided by the authors and unedited

Contents

1 Latent Diffusion Models Setup **2**

1.1 Forward Diffusion Process 2

1.2 Reverse Diffusion Process 2

2 Training and Inference Parameters and Procedure **2**

3 CytoData Labelling Procedure **3**

4 Authenticity Test Setup **3**

5 Psychometric Analysis Details **3**

6 Anomaly Detection Details **4**

7 Ablation Studies on Weighting Functions and Pruning Hyperparameters **4**

8 Generated Images **5**

9 Labeller Confidence Analysis **5**

10 High-Level Explanation of Model Prediction **6**

11 Reduced-Bin Psychometric Analysis **7**

1 Latent Diffusion Models Setup

Generative models have seen significant advancements in recent years, with diffusion models emerging as a leading approach for creating high-quality data samples [37, 61, 62, 63, 64]. These models draw inspiration from thermodynamic diffusion processes [65] and have demonstrated impressive results in various tasks, such as image generation, inpainting, and super-resolution [66, 37, 67, 68, 69]. Diffusion models operate on the principle of defining a forward diffusion process, which gradually adds noise to the data, converting it into a noise-like distribution. The model then learns a reverse process to denoise the data, effectively reconstructing the original data distribution [65, 62]. In latent diffusion models, data $\boldsymbol{x}$ is first encoded into a latent space using an encoder, typically a Variational Autoencoder (VAE), resulting in latent variables $\boldsymbol{z} = \mathcal{E}(\boldsymbol{x})$ with $\boldsymbol{z} \sim q(\boldsymbol{z})$ [70, 37, 71]. The forward and reverse diffusion processes are subsequently applied to these latent variables.

1.1 Forward Diffusion Process

The forward diffusion process is formulated as a Markov chain, wherein Gaussian noise is incrementally added to the latent variables over T steps. Let $\boldsymbol{z}_0$ denote a variable from the encoded (latent) data distribution. The forward process generates a sequence of latent variables $\boldsymbol{z}_1, \boldsymbol{z}_2, \ldots, \boldsymbol{z}_T$ by successively introducing Gaussian noise, with distributions given by

$$q(\boldsymbol{z}_t|\boldsymbol{z}_{t-1}) = \mathcal{N}(\boldsymbol{z}_t; \sqrt{\alpha_t}\boldsymbol{z}_{t-1}, (1-\alpha_t)\boldsymbol{I})\,, \tag{1}$$

where $\alpha_t \in (0, 1)$ gives the variance schedule [65]. The marginal distribution of $\boldsymbol{z}_t$ given $\boldsymbol{z}_0$ is

$$q(\boldsymbol{z}_t|\boldsymbol{z}_0) = \mathcal{N}(\boldsymbol{z}_t; \sqrt{\bar{\alpha}_t}\boldsymbol{z}_0, (1-\bar{\alpha}_t)\boldsymbol{I})\,, \tag{2}$$

with $\bar{\alpha}_t = \prod_{s=1}^{t} \alpha_s$. We will also write $\boldsymbol{z}_t = \sqrt{\bar{\alpha}_t}\boldsymbol{z}_0 + \sqrt{1-\bar{\alpha}_t}\boldsymbol{\epsilon}$, where $\boldsymbol{\epsilon} \sim \mathcal{N}(0, \boldsymbol{I})$.

1.2 Reverse Diffusion Process

The reverse diffusion process seeks to reconstruct the latent variable by reversing the forward process. This process is parameterised by a learnable set of parameters θ, and is conditioned on some context c. We take it to be

$$p_\theta(\boldsymbol{z}_{t-1}|\boldsymbol{z}_t, c) = \mathcal{N}(\boldsymbol{z}_{t-1}; \boldsymbol{\mu}_\theta(\boldsymbol{z}_t, t, c), (1-\alpha_t)\boldsymbol{I})\,, \tag{3}$$

where the mean $\boldsymbol{\mu}_\theta(\boldsymbol{z}_t, t, c)$ is given by

$$\boldsymbol{\mu}_\theta(\boldsymbol{z}_t, t, c) = \frac{1}{\sqrt{\alpha_t}}\left(\boldsymbol{z}_t - \frac{1-\alpha_t}{\sqrt{1-\bar{\alpha}_t}}\boldsymbol{\epsilon}_\theta(\boldsymbol{z}_t, t, c)\right)\,, \tag{4}$$

and where $\boldsymbol{\epsilon}_\theta(\boldsymbol{z}_t, t, c)$ is learned to approximate $\boldsymbol{\epsilon}$ [62].

The training objective for diffusion models is based on the variational lower bound (VLB) of the negative log-likelihood of the data. However, it was shown in [62] that a simpler objective could be used, which measures the difference between the true noise added and that predicted by the model

$$\hat{c} = \underset{c}{\operatorname{argmin}}\, \mathcal{L}_{\text{diffusion}}(c)\,, \tag{5}$$

where $\boldsymbol{\epsilon} \sim \mathcal{N}(0, \boldsymbol{I})$, $t \sim \mathcal{U}[1, T]$ and $w_t = \min\{\frac{5}{\text{SNR}(t)}, 1\}$ is a time-dependent weighting function that balances the importance of different timesteps during training [72, 62].

2 Training and Inference Parameters and Procedure

We applied various data augmentations, including random diagonal flips, random rotation (with angles uniformly sampled between 0 and 359 degrees), colour jitter (brightness = 0.25, contrast = 0.25, saturation = 0.25, hue = 0.125), and Mixup [73] ($\alpha = 0.3$) applied to the conditioning instead of the target and RandAugment with default parameters [74]. We used an AdamW optimiser ($\beta_1 = 0.9$, $\beta_2 = 0.999$, $\epsilon = 10^{-8}$, weight decay 0.01), mixed precision training (fp16), and an exponential moving average of 0.9999. All images were resized to 360×360 pixels.

During inference, we applied the same data augmentations used in training, excluding Mixup. Leveraging the fact that white blood cells are typically centred in our images, and to mitigate the effects of augmentations that corrupt the outer parts of the image, we calculated the inference error only for pixels within a radius of 20 from the centre of the image in our latent space, which has dimensions of $45 \times 45 \times 4$.

For comparisons with EfficientNetV2-M [75] and ViT-B/16 [76], pre-trained on ImageNet [32], we maintained consistent data augmentation techniques and optimiser settings. However, to accommodate the ViT-B/16 model's input size requirements of 384×384 pixels, we first resized the images to match our diffusion model's input size and then further resized them to 384×384 pixels. For these comparison models, we used a batch size of 16, a learning rate of 10^{-4}, and trained for 50 epochs. The model checkpoint with the highest validation accuracy was selected for testing.

3 CytoData Labelling Procedure

CytoData's labelling process involved six haematology experts with {34, 25, 15, 12, 6, 5} years of experience in blood microscopy. Each image was labelled by at least three experts, with 200 images being labelled by all six experts. The experts assigned one of ten classes (basophil, blast, lymphocyte, monocyte, neutrophil, erythroblast, eosinophil, immature granulocyte, platelet or artefact) and indicated their confidence level (High, Moderate, Low or No Confidence) for each prediction. In cases where a majority classification could not be established, two senior experts jointly reviewed the contentious images and determined the final classification.

The experts demonstrated an average inter-rater agreement with a Cohen's κ of 0.81 (see Extended Data Figure 1a), comparable to the $\kappa = 0.84$ reported by Matek et al. [77] and consistent with other studies examining inter-observer reliability in blood cell analysis [78, 79].

The ten-class structure was developed through consultation with haematology experts to balance clinical relevance with practical considerations. Some cell types were merged: immature granulocytes (promyelocytes, myelocytes, and metamyelocytes) were combined into a single class as they share key morphological features, represent a continuous maturation spectrum, and their individual presence often indicates similar underlying conditions. Similarly, neutrophilic bands and segments were merged into a single neutrophil class, reflecting current clinical practice where this distinction is rarely requested.

4 Authenticity Test Setup

We fine-tuned the model on the combined PBC and Bodzas datasets, with all images resized to 512×512 pixels. From this combined dataset, we randomly selected 1,400 images for validation, with the remaining images allocated to the training set. The model was trained for 44,000 steps, employing a limited augmentation strategy that included only random vertical and horizontal flips.

Following the training phase, we generated a set of 144 synthetic images spanning all the classes using 50 inference steps and a guidance scale of 5, where the guidance scale controls the trade-off between image quality and adherence to the class condition [80]. Each image was conditioned on one of nine blood cell types: basophil, blast, lymphocyte, monocyte, neutrophil, erythroblast, eosinophil, immature granulocyte, and platelet. For the Bodzas dataset, we merged the neutrophil segment and neutrophil band classes into a single neutrophil class and created a blast class, which comprised both lymphoblasts and myeloblasts. To create a balanced dataset for the authenticity test, we randomly selected an equivalent set of 144 real images from our validation dataset, resulting in a total pool of 288 images.

5 Psychometric Analysis Details

The "threshold" of the function (m)—an arbitrarily chosen point—and its width (w) are ordinarily measures of performance relative to a ground truth signal strength. Since there is no ground truth here, the goodness of fit of the function to the data, and the separability of different observers are our measures of the quality of the uncertainty. The psychometric function is then defined as

$$\psi(x; m, w, \gamma, \lambda) = \lambda + (1 - \lambda - \gamma) S(x; m, w)\,, \tag{6}$$

where S is a sigmoid, here chosen as the logistic, scaled in the interval [0,1]. We use Bayesian inference, implemented in psignifit (`https://github.com/wichmann-lab/psignifit` under MATLAB 2022b), to obtain parameter estimates, with priors informed by the psychophysical literature [46]. For the threshold, we use a uniform prior over the range of the data with a cosine fall off to 0 over half the range of the data on either side of the maximum and the minimum, expressing a belief that the threshold is located with equal probability within the sampled range and may be up to 50% of the spread of the data outside that range with decreasing probability. For the width, we use a uniform prior between two times the minimal distance of two tested stimulus levels and the range of the stimulus levels with cosine fall offs to 0 at the minimal difference of two stimulus levels and at 3 times the range of the tested stimulus.

Functions were fitted to CytoDiffusion and ViT-B/16 performance with their respective uncertainties using data from the test set; to individual expert performance with mean expert uncertainty; and to individual expert performance with

model uncertainty. To provide sufficient support for each evaluated performance level, uncertainties were discretised into 12 bins.

6 Anomaly Detection Details

We fine-tuned three diffusion models, training each for 140,000 steps on an NVIDIA RTX A5000 GPU. For comparison, we also fine-tuned a Vision Transformer (ViT-B/16) model [76] for each dataset.

For each image, we calculate the error for each class and normalise these errors by dividing by the mean error across all classes for that image. The normality score is then derived by computing the mean difference between the smallest error and all other errors, and subsequently subtracting the mean difference amongst all other errors. A larger score indicates higher confidence, as it suggests a greater distinction between the best-fitting class and the others. For the ViT, we used the logits (before the softmax) instead of the errors.

7 Ablation Studies on Weighting Functions and Pruning Hyperparameters

We conducted extensive ablation studies on different weighting functions and pruning hyperparameters to optimise our model's performance. Our investigations began with the 'Heuristic' and 'Learned' weighting functions described in [22]. The 'Heuristic' function takes the form $\exp(-kt)$, where t is between 0 and 1, and k was initially set to 6 or 7. The 'Learned' function involved dividing the time interval into 20 buckets and fitting a logistic regression function to a small portion of the data to learn the weights.

Our findings revealed that whilst the median error scaled exponentially with time (with $k = 5.5$ in our case, which performed better than 6 or 7), the standard deviation of the error did not follow this pattern. Instead, it formed a curve similar to a negative quadratic function. Given that our primary concern is relative differences rather than absolute values, we focused on this aspect. Consequently, we fitted a fourth-degree polynomial to the median of the standard deviation of the squared errors using validation data from the efficiency experiment with 20 images per class and used the reciprocal of this as our weighting function. The coefficients of our polynomial function are

$$f(t) = 0.927 + 9.837t - 4.684t^2 + 6.019t^3 - 9.340t^4 \,, \tag{7}$$

and this weighting function was used consistently for all experiments presented in this paper. Empirically, this approach outperformed even the fitted 'Learned' logistic regression when using 240 images to train the logistic regression. Notably, our method generalised well, performing effectively on other datasets for which it was not specifically fitted.

We also explored alternative weighting functions. One approach involved calculating the error for all conditions c for one ϵ and t, normalising it to mean 0 and standard deviation 1, then drawing new ϵ and t, normalising to $\mathcal{N}(0, 1)$ etc. and taking the mean of all normalised errors. Another method ranked the errors, assigning a value of 1 to the lowest scoring error, 2 to the next lowest, and so on, before averaging across all trials. We also tested uniform weighting and the SNR weighting used for training: $\min\{\frac{5}{\text{SNR}(t)}, 1\}$.

To evaluate these weighting functions, we conducted ablation studies where we trained a model as described in the "Efficiency in Low-Data Regimes" section, with 20 images per class. During inference, we used {1, 5, 10, 20, 50, 100, 250, 500, 1000} trials per class without any pruning for each weighting function. Supplementary Table 1 presents our results, showing the accuracy after 1000 trials and the mean accuracy across all trials. Our custom weighting function performed best, with the logistic regression and the normalised $\mathcal{N}(0, 1)$ approaches following closely behind.

Supplementary Table 1: Accuracy results for different weighting strategies.

Weighting Strategy	Accuracy after 1000 trials	Mean Accuracy
Custom polynomial	0.9504	0.8805
Logistic regression	0.9458	0.8761
Normalised $\mathcal{N}(0, 1)$	0.9504	0.8743
Uniform	0.9371	0.8704
SNR	0.9371	0.8704
Ranking	0.9467	0.8689
$\exp(-5.5t)$	0.9287	0.8642

We further investigated the impact of the p-value threshold for our pruning and the number of trials to conduct before initiating the Student's t-test to determine if any class should be pruned. Supplementary Figure 1 illustrates these results, showing the mean and standard deviation across all weighting functions combined.

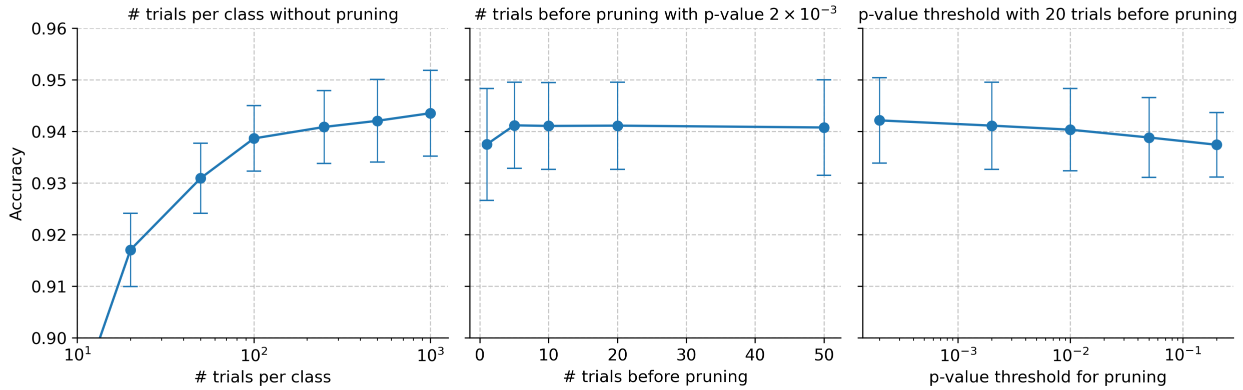

Supplementary Figure 1: The plots show the impact of key hyperparameters on the final accuracy of our diffusion classifier when trained on 20 images per class on the PBC dataset and evaluated on the test set. For each point on the graphs, the statistics were derived by running the model with the 7 different weighting functions shown in Supplementary Table 1. Data points represent the mean accuracy across these 7 replicates, and the vertical error bars show the standard deviation. No statistical tests were performed to compare the data points within these plots; the figure illustrates performance trends when varying the model's hyperparameters. The paired Student's t-test mentioned in the Methods is an internal part of the model's pruning algorithm, not an analysis performed on the data shown here. (Left) Model accuracy as a function of the number of scoring trials per class, with the pruning mechanism disabled. (Middle) The effect of delaying the onset of pruning, shown as the number of trials required before pruning can begin. A fixed p-value threshold of 2×10^3 was used for the internal pruning t-test. (Right) The effect of varying the p-value threshold used for the internal pruning t-test. Pruning was initiated after a fixed 20 trials.

The left panel of Supplementary Figure 1 demonstrates that performance continues to improve with the number of trials per class, even beyond 1000 trials (no pruning was used here). The middle panel shows the effect of delaying the start of pruning until a certain number of trials have been conducted (to avoid prematurely pruning the correct class). Here, we used a fixed p-value threshold of 2×10^{-3}. We observe that as long as we use ≥ 5 steps, the performance is not significantly affected.

The right panel illustrates the impact of the p-value threshold when pruning begins after 20 steps. As expected, performance improves with lower thresholds, approaching the theoretical maximum (the highest value in the left panel) when a value of 2×10^{-4} is used.

8 Generated Images

The images presented in Supplementary Figure 2 showcase the capability of our fine-tuned diffusion model to generate highly realistic synthetic blood cell images. Each image in the grid represents one blood cell class. These synthetic images were part of the dataset used in the authenticity test described in the main text, where expert haematologists were unable to reliably distinguish them from real blood cell images.

9 Labeller Confidence Analysis

To provide insight into the consistency of labeller confidence across different experts, we analysed the agreement between labellers when assessing the same images. Supplementary Figure 3 presents a confusion matrix of labeller confidence scores based on a subset of our dataset. For this analysis, we used a set of 200 images that were independently labelled by all six expert haematologists in our study. We then evaluated their confidence levels pairwise, resulting in 30 comparisons per image. The matrix reveals a strong tendency for labellers to agree on high-confidence assessments, as evidenced by the large number (2,556) in the top-left cell. However, there is also notable disagreement in some cases, particularly between high and moderate confidence levels. Interestingly, instances of 'No Confidence' are relatively rare and often do not align with other labellers' assessments. This could indicate that factors leading to a complete lack of confidence are highly subjective or dependent on individual expertise. These findings underscore the complexity of expert assessment in haematological image analysis and highlight the potential value of incorporating confidence measures in models to go beyond expert human behaviour.

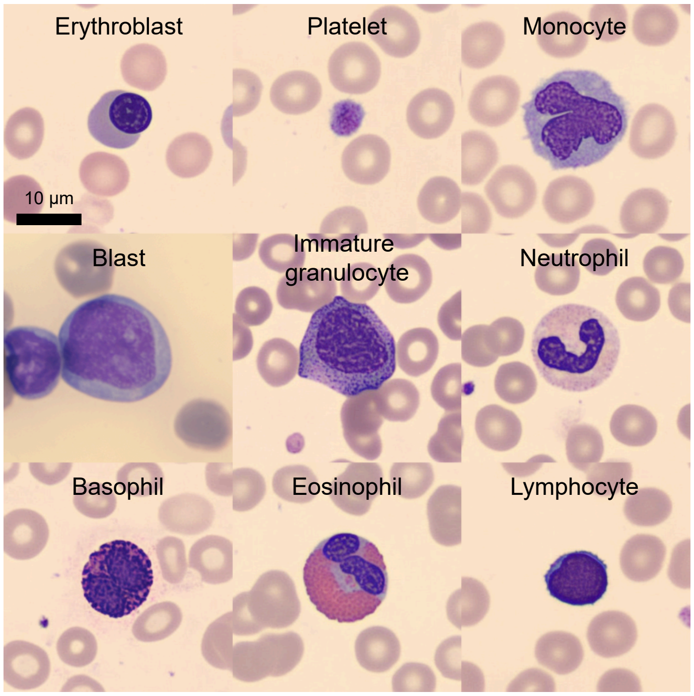

Supplementary Figure 2: Synthetic blood cell images generated by our fine-tuned diffusion model. The figure displays one synthetic image for each of the nine blood cell classes. These images demonstrate the model's ability to capture distinct morphological features of each cell type.

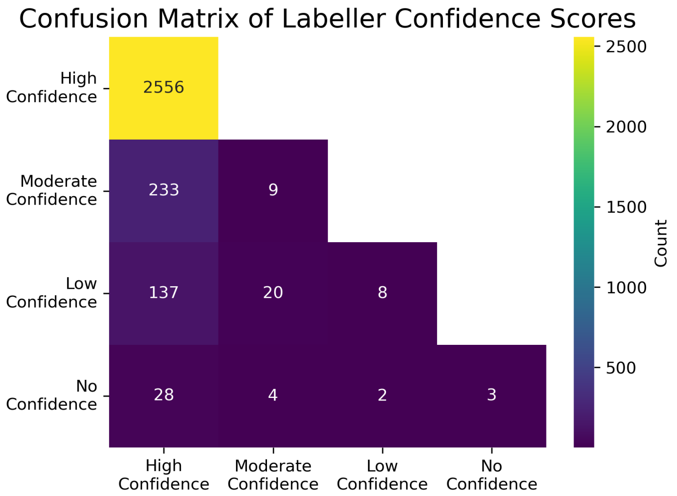

Supplementary Figure 3: Confusion matrix of labeller confidence scores. The matrix shows the frequency of agreement and disagreement between different labellers' confidence levels when assessing the same 200 images. Each cell represents the count of pairwise comparisons (out of 3,000 total comparisons) where the confidence levels of two labellers aligned as indicated. The diagonal represents agreement, whilst off-diagonal elements indicate discrepancies in confidence assessments between labellers.

10 High-Level Explanation of Model Prediction

For an intuitive explanation of how our model makes its predictions, we refer to Supplementary Figure 4. This figure presents a toy example demonstrating how the model determines whether an original image depicts a kitten or a puppy.

The process begins by adding noise to the original image. This noisy image is then fed through the model twice: once with the condition that it is a kitten (that is, the model is told that it is an image of a kitten), and once with the condition that it is a puppy. For each condition, the model predicts how to transform the noisy image into an image of either a kitten or a puppy, respectively. The model then compares the original image with the created images for the different classes. The class that produces the image most closely matching the original is selected as the model's prediction.

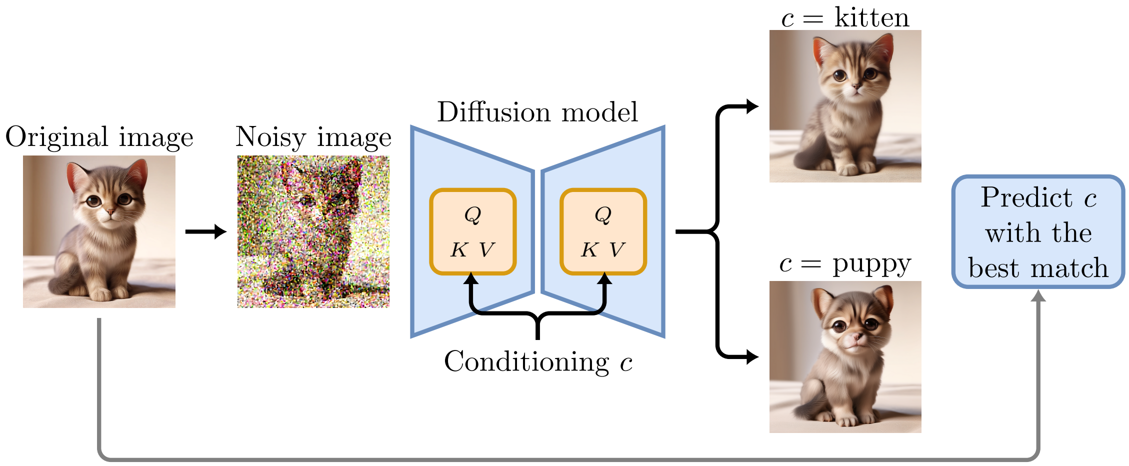

Supplementary Figure 4: High-level overview of how the model predicts a class.

11 Reduced-Bin Psychometric Analysis

We repeated the Bayesian psychometric analyses presented in the main manuscript (Figure 2a-d) using a reduced number of bins (4 instead of 12). Supplementary Figure 5 shows similar sigmoid relationships between confidence and accuracy for CytoDiffusion. The ViT-B/16 model again demonstrates non-monotonic behaviour at higher confidence levels. Individual expert performance assessed via CytoDiffusion or consensus expert confidence retains the previously observed pattern, with CytoDiffusion's confidence providing clearer discrimination of expert performance.

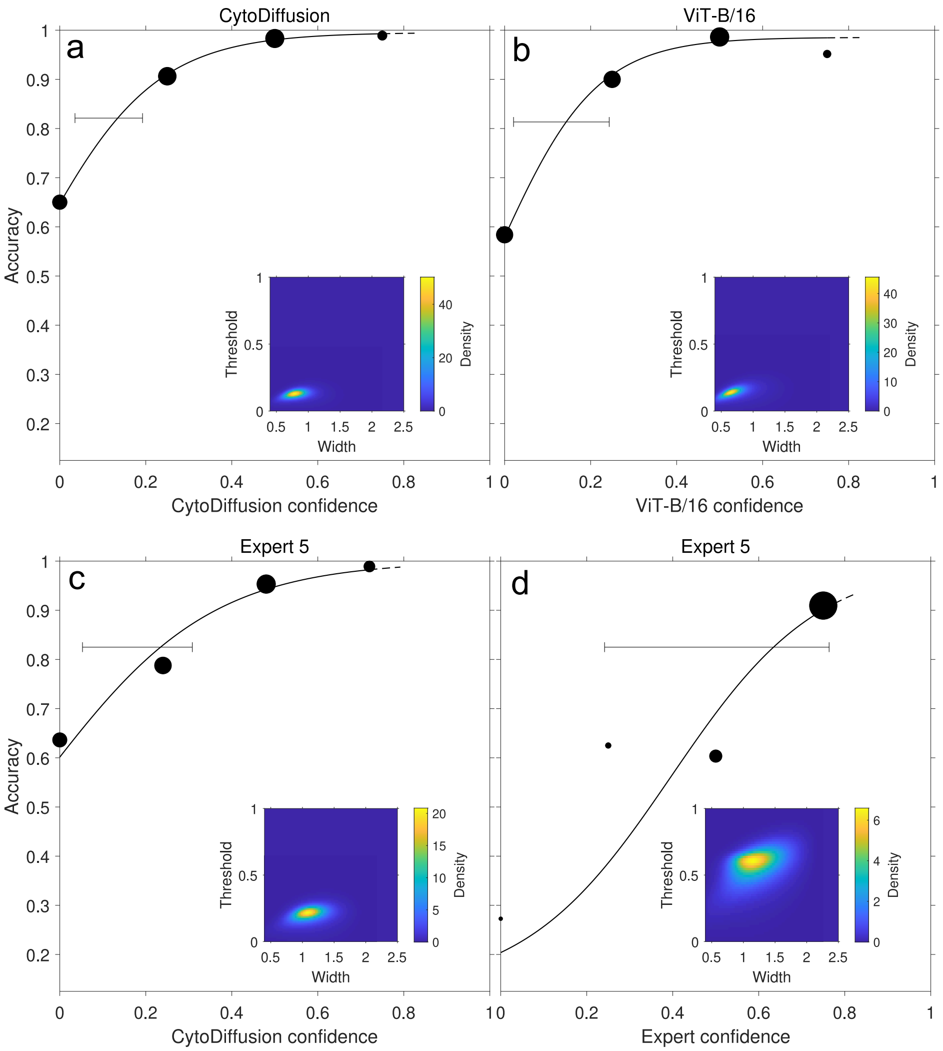

Supplementary Figure 5: Performance was evaluated on our custom CytoData test set ($n = 1,000$ images). (a-d) Psychometric functions showing accuracy as a function of a discriminability index, using a reduced number of four confidence bins. In these panels, data points (black circles) represent the mean accuracy for images within each bin, and their size is proportional to the number of trials in that bin. The solid black line is the maximum-likelihood psychometric function fit to the data. The horizontal black error bar on the curve indicates the 95 per cent credibility interval for the function's threshold, estimated at 80% accuracy (unscaled by lapse and guess rate). Inset plots show the joint posterior probability density for the psychometric function's parameters, width and threshold. (a) Psychometric function for CytoDiffusion, with its own confidence score as the discriminability index. (b) Psychometric function for the ViT-B/16 model, with its own confidence score as the discriminability index. (c) Psychometric function for a representative human expert (Expert 5), using CytoDiffusion's confidence score as the discriminability index. (d) Psychometric function for the same expert (Expert 5), using expert confidence as the discriminability index.

Supplementary Table 2: Dataset Usage Across Experimental Tasks. ✓ indicates dataset was used for the corresponding task; ✗ indicates dataset was not used.

**Task**	**Raabin-WBC**	**PBC**	**Bodzas**	**CytoData**	**Explanation**
Classification	✓	✓	✓	✓	
Low-data Regime	✓	✓	✓	✓	
Domain Shift	✓	✓	✓	✓	
Anomaly Detection	✗	✓	✓	✓	Raabin-WBC was excluded as it does not contain abnormal cell classes required for this analysis.
Uncertainty Quantification	✗	✗	✗	✓	Only CytoData contains expert confidence scores, making it uniquely suited for comparing human and model uncertainty estimates.
Authenticity Testing	✗	✓	✓	✗	This was conducted as an initial validation experiment on a subset of datasets to establish the model's potential.
Explainability	✗	✓	✗	✗	Explainability analysis was shown as a proof of concept on a single dataset.

References

[1] Barbara J Bain. *Blood cells: a practical guide*. John Wiley & Sons, 2021.

[2] Alexander Kratz, Szu-hee Lee, Gina Zini, Jurgen A. Riedl, Mina Hur, Sam Machin, and the International Council for Standardization in Haematology. Digital morphology analyzers in hematology: Icsh review and recommendations. *International Journal of Laboratory Hematology*, 41(4):437–447, 2019.

[3] Mauro Buttarello and Mario Plebani. Automated blood cell counts: State of the art. *American Journal of Clinical Pathology*, 130(1):104–116, July 2008.

[4] Gert-Jan van de Geijn, Vincent van Rees, Natasja van Pul-Bom, Erwin Birnie, Hans Janssen, Hans Pegels, Marlène Beunis, and Tjin Njo. Leukoflow: Multiparameter extended white blood cell differentiation for routine analysis by flow cytometry. *Cytometry Part A*, 79A(9):694–706, 2011.

[5] G E Metter, B N Nathwani, J S Burke, C D Winberg, R B Mann, M Barcos, C R Kjeldsberg, C C Whitcomb, D O Dixon, and T P Miller. Morphological subclassification of follicular lymphoma: variability of diagnoses among hematopathologists, a collaborative study between the repository center and pathology panel for lymphoma clinical studies. *Journal of Clinical Oncology*, 3(1):25–38, January 1985.

[6] Maíla Claro, Luis Vogado, Rodrigo Veras, André Santana, João Tavares, Justino Santos, and Vinicius Machado. Convolution neural network models for acute leukemia diagnosis. In *2020 international conference on systems, signals and image processing (IWSSIP)*, pages 63–68. IEEE, 2020.

[7] Tatdow Pansombut, Siripen Wikaisuksakul, Kittiya Khongkraphan, and Aniruth Phon-On. Convolutional neural networks for recognition of lymphoblast cell images. *Computational Intelligence and Neuroscience*, 2019(1):7519603, 2019.

[8] Matthias Hehr, Ario Sadafi, Christian Matek, Peter Lienemann, Christian Pohlkamp, Torsten Haferlach, Karsten Spiekermann, and Carsten Marr. Explainable ai identifies diagnostic cells of genetic aml subtypes. *PLOS Digital Health*, 2(3):e0000187, 2023.

[9] Austin H Routt, Natalia Yang, Nathaniel Z Piety, Madeleine Lu, and Sergey S Shevkoplyas. Deep ensemble learning enables highly accurate classification of stored red blood cell morphology. *Scientific Reports*, 13(1):3152, 2023.

[10] Minh Doan, Joseph A Sebastian, Juan C Caicedo, Stefanie Siegert, Aline Roch, Tracey R Turner, Olga Mykhailova, Ruben N Pinto, Claire McQuin, Allen Goodman, et al. Objective assessment of stored blood quality by deep learning. *Proceedings of the National Academy of Sciences*, 117(35):21381–21390, 2020.

[11] Christian Matek, Sebastian Krappe, Christian Münzenmayer, Torsten Haferlach, and Carsten Marr. Highly accurate differentiation of bone marrow cell morphologies using deep neural networks on a large image data set. *Blood, The Journal of the American Society of Hematology*, 138(20):1917–1927, 2021.

[12] Jee Seok Yoon, Kwanseok Oh, Yooseung Shin, Maciej A. Mazurowski, and Heung-Il Suk. Domain generalization for medical image analysis: A review. *Proceedings of the IEEE*, 112(10):1583–1609, 2024.

[13] Pang Wei Koh, Shiori Sagawa, Henrik Marklund, Sang Michael Xie, Marvin Zhang, Akshay Balsubramani, Weihua Hu, Michihiro Yasunaga, Richard Lanas Phillips, Irena Gao, Tony Lee, Etienne David, Ian Stavness, Wei Guo, Berton Earnshaw, Imran Haque, Sara M. Beery, Jure Leskovec, Anshul Kundaje, Emma Pierson, Sergey Levine, Chelsea Finn, and Percy Liang. Wilds: A benchmark of in-the-wild distribution shifts. In *Proceedings of the 38th International Conference on Machine Learning*, page 5637–5664. PMLR, July 2021.

[14] Andreas Holzinger, Georg Langs, Helmut Denk, Kurt Zatloukal, and Heimo Müller. Causability and explainability of artificial intelligence in medicine. *Wiley Interdisciplinary Reviews. Data Mining and Knowledge Discovery*, 9(4):e1312, 2019.

[15] Cynthia Rudin. Stop explaining black box machine learning models for high stakes decisions and use interpretable models instead. *Nature Machine Intelligence*, 1(5):206–215, May 2019.

[16] Amirhossein Kazerouni, Ehsan Khodapanah Aghdam, Moein Heidari, Reza Azad, Mohsen Fayyaz, Ilker Hacihaliloglu, and Dorit Merhof. Diffusion models in medical imaging: A comprehensive survey. *Medical Image Analysis*, 88:102846, August 2023.

[17] Edmon Begoli, Tanmoy Bhattacharya, and Dimitri Kusnezov. The need for uncertainty quantification in machine-assisted medical decision making. *Nature Machine Intelligence*, 1(1):20–23, January 2019.

[18] Rabia Asghar, Sanjay Kumar, Arslan Shaukat, and Paul Hynds. Classification of white blood cells (leucocytes) from blood smear imagery using machine and deep learning models: A global scoping review. *Plos one*, 19(6):e0292026, 2024.

[19] Rakesh Kumar, Pooja Kumbharkar, Sandeep Vanam, and Sanjeev Sharma. Medical images classification using deep learning: a survey. *Multimedia Tools and Applications*, 83(7):19683–19728, 2024.

[20] Alexander Cong Li, Ananya Kumar, and Deepak Pathak. Generative classifiers avoid shortcut solutions. In *ICML 2024 Workshop on Structured Probabilistic Inference & Generative Modeling*, 2024.

[21] Huanran Chen, Yinpeng Dong, Zhengyi Wang, Xiao Yang, Chengqi Duan, Hang Su, and Jun Zhu. Robust classification via a single diffusion model. In *Proceedings of the 41st International Conference on Machine Learning*, volume 235 of *Proceedings of Machine Learning Research*, pages 6643–6665. PMLR, 21–27 Jul 2024.

[22] Kevin Clark and Priyank Jaini. Text-to-image diffusion models are zero shot classifiers. *Advances in Neural Information Processing Systems*, 36, 2024.

[23] Alexander C Li, Mihir Prabhudesai, Shivam Duggal, Ellis Brown, and Deepak Pathak. Your diffusion model is secretly a zero-shot classifier. In *Proceedings of the IEEE/CVF International Conference on Computer Vision*, pages 2206–2217, 2023.

[24] Robin Rombach, Andreas Blattmann, Dominik Lorenz, Patrick Esser, and Björn Ommer. High-resolution image synthesis with latent diffusion models. In *Proceedings of the IEEE/CVF Conference on Computer Vision and Pattern Recognition (CVPR)*, pages 10684–10695, June 2022.

[25] Zahra Mousavi Kouzehkanan, Sepehr Saghari, Sajad Tavakoli, Peyman Rostami, Mohammadjavad Abaszadeh, Farzaneh Mirzadeh, Esmaeil Shahabi Satlsar, Maryam Gheidishahran, Fatemeh Gorgi, Saeed Mohammadi, and Reshad Hosseini. A large dataset of white blood cells containing cell locations and types, along with segmented nuclei and cytoplasm. *Scientific Reports*, 12(1):1123, January 2022.

[26] Andrea Acevedo, Anna Merino, Santiago Alférez, Ángel Molina, Laura Boldú, and José Rodellar. A dataset of microscopic peripheral blood cell images for development of automatic recognition systems. *Data in Brief*, 30:105474, June 2020.

[27] Alexandra Bodzas, Pavel Kodytek, and Jan Zidek. A high-resolution large-scale dataset of pathological and normal white blood cells. *Scientific Data*, 10(11):466, July 2023.

[28] Alex Kendall and Yarin Gal. What uncertainties do we need in bayesian deep learning for computer vision? *Advances in neural information processing systems*, 30, 2017.

[29] Yaniv Ovadia, Emily Fertig, Jie Ren, Zachary Nado, D. Sculley, Sebastian Nowozin, Joshua Dillon, Balaji Lakshminarayanan, and Jasper Snoek. Can you trust your model' s uncertainty? evaluating predictive uncertainty under dataset shift. In *Advances in Neural Information Processing Systems*, volume 32. Curran Associates, Inc., 2019.

[30] Ramprasaath R Selvaraju, Michael Cogswell, Abhishek Das, Ramakrishna Vedantam, Devi Parikh, and Dhruv Batra. Grad-cam: Visual explanations from deep networks via gradient-based localization. In *Proceedings of the IEEE international conference on computer vision*, pages 618–626, 2017.

[31] Marco Tulio Ribeiro, Sameer Singh, and Carlos Guestrin. " why should i trust you?" explaining the predictions of any classifier. In *Proceedings of the 22nd ACM SIGKDD international conference on knowledge discovery and data mining*, pages 1135–1144, 2016.

[32] Jia Deng, Wei Dong, Richard Socher, Li-Jia Li, Kai Li, and Li Fei-Fei. Imagenet: A large-scale hierarchical image database. In *2009 IEEE Conference on Computer Vision and Pattern Recognition*, page 248–255, June 2009.

[33] Arundhati S Shanbhag, Brian B Moser, Tobias C Nauen, Stanislav Frolov, Federico Raue, and Andreas Dengel. Just leaf it: Accelerating diffusion classifiers with hierarchical class pruning. *arXiv preprint arXiv:2411.12073*, 2024.

[34] Guilherme Pombo, Robert Gray, M Jorge Cardoso, Sebastien Ourselin, Geraint Rees, John Ashburner, and Parashkev Nachev. Equitable modelling of brain imaging by counterfactual augmentation with morphologically constrained 3d deep generative models. *Medical Image Analysis*, 84:102723, 2023.

[35] Edward Paulson. A sequential procedure for selecting the population with the largest mean from k normal populations. *The Annals of Mathematical Statistics*, pages 174–180, 1964.

[36] Eyal Even-Dar, Shie Mannor, and Yishay Mansour. Pac bounds for multi-armed bandit and markov decision processes. In *Computational Learning Theory: 15th Annual Conference on Computational Learning Theory, COLT 2002 Sydney, Australia, July 8–10, 2002 Proceedings 15*, pages 255–270. Springer, 2002.

[37] Robin Rombach, Andreas Blattmann, Dominik Lorenz, Patrick Esser, and Björn Ommer. High-resolution image synthesis with latent diffusion models. In *Proceedings of the IEEE/CVF conference on computer vision and pattern recognition*, pages 10684–10695, 2022.

[38] Hüseyin Fırat. Classification of microscopic peripheral blood cell images using multibranch lightweight cnn-based model. *Neural Computing and Applications*, 36(4):1599–1620, February 2024.

[39] Mohamad Abou Ali, Fadi Dornaika, and Ignacio Arganda-Carreras. Blood cell revolution: Unveiling 11 distinct types with 'naturalize' augmentation. *Algorithms*, 16(1212):562, December 2023.

[40] Gabriel Kalweit, Anusha Klett, Mehdi Naouar, Jens Rahnfeld, Yannick Vogt, Diana Laura Infante Ramirez, Rebecca Berger, Jesus Duque Afonso, Tanja Nicole Hartmann, Marie Follo, et al. Unsupervised feature extraction from a foundation model zoo for cell similarity search in oncological microscopy across devices. In *ICML Workshop on Foundation Models in the Wild*, 2024.

[41] Albert Garcia Llagostera. Developing a scalable and privacy-preserving deep learning model for the classification of peripheral blood cell images. *Universitat Oberta de Catalunya (UOC)*, 2024.

[42] Ruitao Zhang, Xueying Han, Zhengyang Lei, Chenyao Jiang, Ijaz Gul, Qiuyue Hu, Shiyao Zhai, Hong Liu, Lijin Lian, Ying Liu, Yongbing Zhang, Yuhan Dong, Can Yang Zhang, Tsz Kwan Lam, Yuxing Han, Dongmei Yu, Jin Zhou, and Peiwu Qin. Rcmnet: A deep learning model assists car-t therapy for leukemia. *Computers in Biology and Medicine*, 150:106084, November 2022.

[43] Fei Long, Jing-Jie Peng, Weitao Song, Xiaobo Xia, and Jun Sang. Bloodcaps: A capsule network based model for the multiclassification of human peripheral blood cells. *Computer Methods and Programs in Biomedicine*, 202:105972, April 2021.

[44] Rufus Rubin, S. M. Anzar, Alavikunhu Panthakkan, and Wathiq Mansoor. Transforming healthcare: Raabin white blood cell classification with deep vision transformer. In *2023 6th International Conference on Signal Processing and Information Security, ICSPIS 2023*, page 212–217. Institute of Electrical and Electronics Engineers Inc., 2023.

[45] George A. Gescheider. *Psychophysics: The Fundamentals*. Psychology Press, New York, 3 edition, May 1997.

[46] Heiko H Schütt, Stefan Harmeling, Jakob H Macke, and Felix A Wichmann. Painfree and accurate bayesian estimation of psychometric functions for (potentially) overdispersed data. *Vision research*, 122:105–123, 2016.

[47] Yee-Haur Mah, Rolf Jager, Christopher Kennard, Masud Husain, and Parashkev Nachev. A new method for automated high-dimensional lesion segmentation evaluated in vascular injury and applied to the human occipital lobe. *Cortex*, 56:51–63, 2014. The clinical neuroanatomy of the occipital lobes.

[48] Konrad Rieck and Pavel Laskov. Detecting unknown network attacks using language models. In *Detection of Intrusions and Malware & Vulnerability Assessment: Third International Conference, DIMVA 2006, Berlin, Germany, July 13-14, 2006. Proceedings 3*, pages 74–90. Springer, 2006.

[49] Zahra Mousavi Kouzehkanan, Sepehr Saghari, Sajad Tavakoli, Peyman Rostami, Mohammadjavad Abaszadeh, Farzaneh Mirzadeh, Esmaeil Shahabi Satlsar, Maryam Gheidishahran, Fatemeh Gorgi, Saeed Mohammadi, and Reshad Hosseini. A large dataset of white blood cells containing cell locations and types, along with segmented nuclei and cytoplasm. *Scientific Reports*, 12(11):1123, January 2022.

[50] Seyed Hamid Rezatofighi and Hamid Soltanian-Zadeh. Automatic recognition of five types of white blood cells in peripheral blood. *Computerized Medical Imaging and Graphics*, 35(4):333–343, 2011.

[51] Chongchong Li and Yuting Liu. Improved generalization of white blood cell classification by learnable illumination intensity invariant layer. *IEEE Signal Processing Letters*, 31:176–180, 2024.

[52] Satoshi Tsutsui, Zhengyang Su, and Bihan Wen. Benchmarking white blood cell classification under domain shift. In *ICASSP 2023 - 2023 IEEE International Conference on Acoustics, Speech and Signal Processing (ICASSP)*, page 1–5, June 2023.

[53] Simon Deltadahl, Michael Roberts, Parashkev Nachev, et al. CambridgeCIA/CytoDiffusion. *Zenodo* DOI: `https://doi.org/10.5281/zenodo.14825812`.

[54] Sajad Tavakoli, Ali Ghaffari, Zahra Mousavi Kouzehkanan, and Reshad Hosseini. New segmentation and feature extraction algorithm for classification of white blood cells in peripheral smear images. *Scientific Reports*, 11(1):19428, September 2021.

[55] Hua Chen, Juan Liu, Chunbing Hua, Jing Feng, Baochuan Pang, Dehua Cao, and Cheng Li. Accurate classification of white blood cells by coupling pre-trained resnet and densenet with scam mechanism. *BMC Bioinformatics*, 23:282, July 2022.

[56] Jiang L, Tang C, and Zhou H. White blood cell classification via a discriminative region detection assisted feature aggregation network. *Biomedical optics express*, 13(10), September 2022.

[57] Eduardo Rivas-Posada and Mario I. Chacon-Murguia. Automatic base-model selection for white blood cell image classification using meta-learning. *Computers in Biology and Medicine*, 163:107200, September 2023.

[58] Ferhat Ucar. Deep learning approach to cell classificatio in human peripheral blood. *2020 5th International Conference on Computer Science and Engineering (UBMK)*, page 383–387, September 2020.

[59] Priyanka Rastogi, Kavita Khanna, and Vijendra Singh. Leufeatx: Deep learning–based feature extractor for the diagnosis of acute leukemia from microscopic images of peripheral blood smear. *Computers in Biology and Medicine*, 142:105236, March 2022.

[60] Sudhakar Tummala and Anil K. Suresh. Few-shot learning using explainable siamese twin network for the automated classification of blood cells. *Medical & Biological Engineering & Computing*, 61(6):1549–1563, June 2023.

[61] Minshuo Chen, Song Mei, Jianqing Fan, and Mengdi Wang. An overview of diffusion models: Applications, guided generation, statistical rates and optimization. *arXiv preprint arXiv:2404.07771*, 2024.

[62] Jonathan Ho, Ajay Jain, and Pieter Abbeel. Denoising diffusion probabilistic models. *Advances in neural information processing systems*, 33:6840–6851, 2020.

[63] Yang Song, Jascha Sohl-Dickstein, Diederik P. Kingma, Abhishek Kumar, Stefano Ermon, and Ben Poole. Score-based generative modeling through stochastic differential equations. In *International Conference on Learning Representations (ICLR)*, 2021.

[64] Yang Song and Stefano Ermon. Improved techniques for training score-based generative models. In *Advances in Neural Information Processing Systems*, volume 33, page 12438–12448. Curran Associates, Inc., 2020.

[65] Jascha Sohl-Dickstein, Eric Weiss, Niru Maheswaranathan, and Surya Ganguli. Deep unsupervised learning using nonequilibrium thermodynamics. In *International conference on machine learning*, pages 2256–2265. PMLR, 2015.

[66] Chitwan Saharia, William Chan, Saurabh Saxena, Lala Li, Jay Whang, Emily L Denton, Kamyar Ghasemipour, Raphael Gontijo Lopes, Burcu Karagol Ayan, Tim Salimans, et al. Photorealistic text-to-image diffusion models with deep language understanding. *Advances in neural information processing systems*, 35:36479–36494, 2022.

[67] Chitwan Saharia, William Chan, Huiwen Chang, Chris Lee, Jonathan Ho, Tim Salimans, David Fleet, and Mohammad Norouzi. Palette: Image-to-image diffusion models. In *ACM SIGGRAPH 2022 conference proceedings*, pages 1–10, 2022.

[68] Andreas Lugmayr, Martin Danelljan, Andres Romero, Fisher Yu, Radu Timofte, and Luc Van Gool. Repaint: Inpainting using denoising diffusion probabilistic models. In *Proceedings of the IEEE/CVF conference on computer vision and pattern recognition*, pages 11461–11471, 2022.

[69] Chitwan Saharia, Jonathan Ho, William Chan, Tim Salimans, David J Fleet, and Mohammad Norouzi. Image super-resolution via iterative refinement. *IEEE transactions on pattern analysis and machine intelligence*, 45(4):4713–4726, 2022.

[70] Aaron Van Den Oord, Oriol Vinyals, et al. Neural discrete representation learning. *Advances in neural information processing systems*, 30, 2017.

[71] Diederik P. Kingma and Max Welling. Auto-encoding variational bayes. In *2nd International Conference on Learning Representations, ICLR, Banff, Canada, April 14-16*, 2014.

[72] Tiankai Hang, Shuyang Gu, Chen Li, Jianmin Bao, Dong Chen, Han Hu, Xin Geng, and Baining Guo. Efficient diffusion training via min-snr weighting strategy. In *Proceedings of the IEEE/CVF International Conference on Computer Vision*, pages 7441–7451, 2023.

[73] Hongyi Zhang, Moustapha Cissé, Yann N Dauphin, and David Lopez-Paz. mixup: Beyond empirical risk minimization. In *International Conference on Learning Representations*, 2018.

[74] Ekin D Cubuk, Barret Zoph, Jonathon Shlens, and Quoc V Le. Randaugment: Practical automated data augmentation with a reduced search space. In *Proceedings of the IEEE/CVF conference on computer vision and pattern recognition workshops*, pages 702–703, 2020.

[75] Mingxing Tan and Quoc Le. Efficientnetv2: Smaller models and faster training. In *International conference on machine learning*, pages 10096–10106. PMLR, 2021.

[76] Alexey Dosovitskiy, Lucas Beyer, Alexander Kolesnikov, Dirk Weissenborn, Xiaohua Zhai, Thomas Unterthiner, Mostafa Dehghani, Matthias Minderer, Georg Heigold, Sylvain Gelly, Jakob Uszkoreit, and Neil Houlsby. An image is worth 16x16 words: Transformers for image recognition at scale, 2021.

[77] Christian Matek, Simone Schwarz, Karsten Spiekermann, and Carsten Marr. Human-level recognition of blast cells in acute myeloid leukaemia with convolutional neural networks. *Nature Machine Intelligence*, 1(11):538–544, November 2019.

[78] P Font, J Loscertales, C Benavente, A Bermejo, M Callejas, L Garcia-Alonso, A Garcia-Marcilla, S Gil, M Lopez-Rubio, E Martin, et al. Inter-observer variance with the diagnosis of myelodysplastic syndromes (mds) following the 2008 who classification. *Annals of hematology*, 92:19–24, 2013.

[79] Patricia Font, Javier Loscertales, Carlos Soto, Pilar Ricard, Carolina Muñoz Novas, Estela Martín-Clavero, Montserrat López-Rubio, Luis Garcia-Alonso, Marta Callejas, Alfredo Bermejo, et al. Interobserver variance in myelodysplastic syndromes with less than 5% bone marrow blasts: unilineage vs. multilineage dysplasia and reproducibility of the threshold of 2% blasts. *Annals of hematology*, 94:565–573, 2015.

[80] Jonathan Ho and Tim Salimans. Classifier-free diffusion guidance. *arXiv preprint arXiv:2207.12598*, 2022.
